# Transcriptome Landscape of Cancer‐Associated Fibroblasts in Human PDAC

**DOI:** 10.1002/advs.202415196

**Published:** 2025-02-28

**Authors:** Mengyu Tao, Wenting Liu, Jianhua Chen, Rujiao Liu, Jianling Zou, Bo Yu, Chenchen Wang, Mingzhu Huang, Qingjian Chen, Zhe Zhang, Zhiyu Chen, Haoyu Sun, Cheng Zhou, Shuguang Tan, Yuxuan Zheng, Hongxia Wang

**Affiliations:** ^1^ Department of Oncology Shanghai General Hospital Shanghai Jiaotong University School of Medicine Shanghai 200800 P. R. China; ^2^ Department of Medical Oncology Fudan University Shanghai Cancer Center Shanghai 200032 P. R. China; ^3^ Department of Oncology Shanghai Medical College Fudan University Shanghai 200032 P. R. China; ^4^ Department of Immunology School of Basic Medical Sciences Fudan University Shanghai 200032 China; ^5^ Department of Radiation Oncology Nanfang Hospital Southern Medical University Guangzhou 510515 P. R. China; ^6^ The Second Affiliated Hospital Zhejiang University School of Medicine Hangzhou 310009 China; ^7^ Human Phenome Institute Minhang Hosptial Fudan University Shanghai 201203 P. R. China

**Keywords:** AP‐1 family, CAF, CDCP1, heterogeneity, PDAC

## Abstract

Cancer‐associated fibroblasts (CAFs) play a crucial role in the progression of pancreatic ductal adenocarcinoma (PDAC). Here, integrated single‐cell RNA sequencing analysis is utilized to comprehensively map CAFs in the human PDAC tumor microenvironment (TME). Normal fibroblasts (NFs) and nine distinct CAF subtypes are identified including newly identified CAF subtypes, CDCP1^+^FTL^+^ CAFs, transitional CAFs (tCAFs), interferon simulated genes (ISG)^+^ myofibroblastic CAFs (myCAFs), and proliferative CAFs (pCAFs). CDCP1^+^FTL^+^ CAFs, pCAFs, and ISG^+^ myCAFs are associated with unfavorable clinical outcomes. CDCP1^+^FTL^+^ CAFs exhibit enhanced glycolysis and iron metabolism, resisting ferroptosis. The antigen‐presenting CAFs (apCAFs) show high heterogeneity, consisting of multiple subtypes expressing distinct immune cell signatures. The CAF subtypes display differentiation plasticity, transitioning from early normal‐like CAFs (nCAFs) to inflammatory CAFs (iCAFs) and myCAFs, ultimately leading to more invasive pCAFs. AP‐1 family members FOS and JUN regulate the malignant phenotype conversion of NFs to nCAFs, while transforming growth factor‐β (TGFβ) and interferon‐γ (IFNγ) signals trigger the interconversion between classic myCAFs and iCAFs, respectively. A close interaction between CAFs and myeloid cells (especially neutrophils) is further observed in PDAC‐TME, mainly mediated by CXCR4‐CXCL12 chemotaxis. This work depicts a detailed CAF map and its dynamic interconvertible shift, providing important insights for combined targeted CAFs therapy.

## Graphical abstract

AP‐1 family members FOS and JUN regulate the malignant phenotype conversion of normal fibroblasts (NFs) to normal‐like cancer‐associated fibroblasts (nCAFs), while transforming growth factor‐β (TGFβ) and interferon‐γ (IFNγ) signals triggers the interconversion between classic myofibroblastic CAFs (myCAFs) and inflammatory CAFs (iCAFs), respectively. CAFs interact with myeloid cells mainly mediated by CXCR4‐CXCL12 chemotaxis. Hypoxia‐induced CDCP1^+^FTL^+^ CAFs exhibit enhanced glycolysis and iron metabolism, resisting ferroptosis.

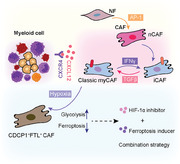

## 1 Introduction

Pancreatic ductal adenocarcinoma (PDAC) is a highly lethal malignancy characterized by poor prognosis, with median survival of ∼6 months and 5‐year survival rate of less than 10%.^[^ ^1^ ^]^ Systemic therapy including chemotherapy and targeted therapy being the mainstay of treatment only slightly prolongs survival.^[^ ^2^ ^]^ Immunotherapies, such as immune checkpoint inhibitors (ICIs) and chimeric antigen receptor T cells (CAR‐T), exhibiting the potential to target tumors precisely, have been unsuccessful in PDAC patients.^[^ ^3^, ^4^ ^]^

The PDAC tumor microenvironment (TME) consists of a complex ecosystem composed of cancer‐associated fibroblasts (CAFs), immune cells, endothelial cells, and others.^[^ ^5^ ^]^ Among them, CAFs represent one of the most prominent and plastic cell types ascribed as key players with pleiotropic protumorigenic functions. CAFs can produce components of the extracellular matrix (ECM), acting as a physical barrier with increased tissue stiffening impairing drug delivery.^[^ ^6^, ^7^, ^8^ ^]^ Meanwhile, CAFs possess the capacity to biochemically stimulate cancer cell proliferation and invasion, drive immunosuppression, as well as modulate the sensitivity to ICIs treatment.^[^ ^9^, ^10^, ^11^, ^12^, ^13^ ^]^ These findings highlight the important role of CAFs for PDAC progression and raise the potential of CAFs as therapeutic targets. However, the prevailing belief has been recently challenged by clinical evidence indicating that therapeutic inhibitors targeting CAFs have demonstrated decreased survival and accelerated disease progression in both mouse models and clinical trials.^[^ ^14^, ^15^, ^16^ ^]^ The disappointing results illustrate the heterogeneity and complexity of CAFs population in the human TME. CAFs co‐evolve with cancer cells adopting diverse phenotypes, displaying diametrically opposite effects either pro‐ or anti‐tumor growth.^[^ ^7^ ^]^ One way to reconcile the contradiction is to develop a precise targeting‐therapy by pinpointing specific protumorigenic CAFs.

Although several cell surface markers such as α‐SMA (α‐smooth muscle actin), FAP (fibroblast activation protein alpha), S100A4/FSP1 (fibroblast‐specific protein 1), platelet‐derived growth factor receptor‐β (PDGFR‐β), and CAV1 (caveolin 1) have been used for labeling and sorting CAFs, none of them reliably identifies all CAFs. Recent advancements in single‐cell RNA sequencing (scRNA‐seq) techniques have enabled the analysis of intra‐tumoral heterogeneity, unveiling the presence of diverse CAF subtypes such as myofibroblastic CAFs (myCAFs), inflammatory CAFs (iCAFs), and antigen‐presenting CAFs (apCAFs).^[^ ^17^ ^]^ These subtypes exhibit reversible cell states that are polarized by their proximity to cancer cells.^[^ ^18^, ^19^, ^20^ ^]^ However, the comprehensive understanding of CAFs’ functional heterogeneity, differentiate plasticity, and potential mutual transformation between different subtypes remains limited.

Here, through integrated scRNA‐seq analysis of ∼12 000 fibroblasts from PDAC tissues and normal pancreas tissues, we charted the complexity of CAFs. The study identified normal fibroblasts (NFs) and nine different subtypes of CAFs, including newly identified CUB domain containing protein 1 (CDCP1)^+^ ferritin light chain (FTL)^+^ CAFs, transitional CAFs (tCAFs), interferon simulated genes (ISG)^+^ myCAFs, and proliferative CAFs (pCAFs). CDCP1^+^FTL^+^ CAFs were mainly enriched in the hypoxic region of tumors and can be induced under hypoxic conditions. These cells exhibited enhanced glycolysis and iron metabolism, and resistance to ferroptosis. The tCAFs subset represented an intermediate state bridging myCAFs and iCAFs conversion. CDCP1^+^FTL^+^ CAFs, pCAFs, and ISG^+^ myCAFs were associated with unfavorable clinical outcomes. The CAF subtypes displayed differentiation plasticity, transitioning from early normal‐like CAFs (nCAFs) to iCAFs and myCAFs, ultimately leading to more invasive pCAFs. AP‐1 family members FOS and JUN regulated the malignant phenotype transformation of NFs to nCAFs, while transforming growth factor‐β (TGFβ) and interferon‐γ (IFNγ) signaling triggered the interconversion between classic myCAF and iCAF, respectively. A close interaction between CAFs and myeloid cells (especially neutrophils) was further observed in PDAC‐TME, mainly mediated by CXCR4‐CXCL12 chemotaxis. Overall, our study provides a comprehensive understanding of CAFs, complements recent research in this field, and offers valuable insights for potential combined therapeutic targeting of CAFs.

## 2 Results

### 2.1 Heterogeneity of the PDAC‐TME at Single‐cell Resolution

**Figure 1:**
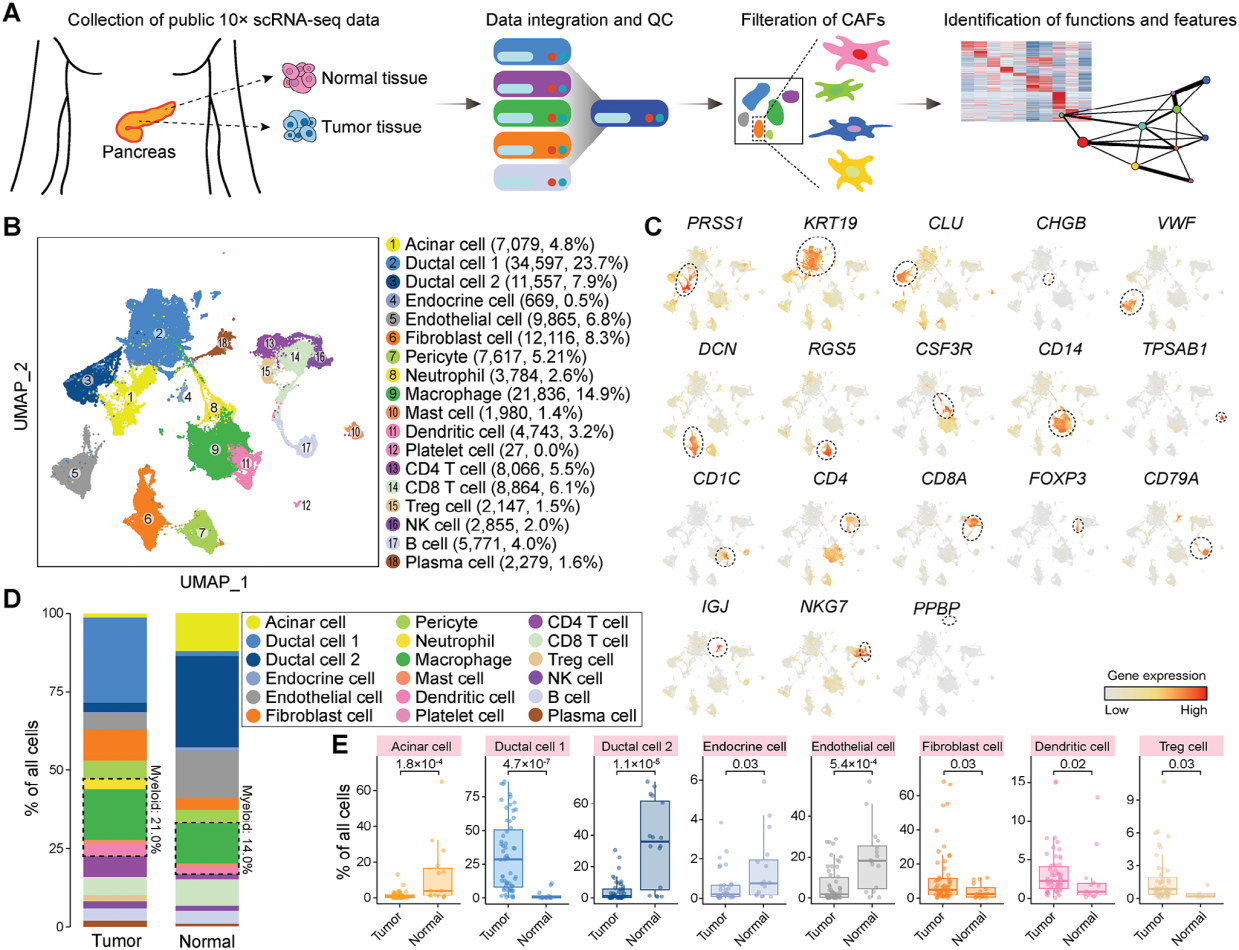
Single‐cell landscape of all cells from tumor and normal tissues. A) Schema of this study. B) UMAP plot showing the clustering of all cells. The dot color indicates cell types. The number and percentage of cells in each cell type are indicated in brackets. C) UMAP plots showing the expression level of marker genes. The corresponding cell type is indicated with dash circles. D) Stacked barplot showing the percentage of distinct cell types among all cells from tumor or normal tissues. The myeloid cell is indicated with dash boxes, and the corresponding percentage is indicated. E) Boxplots showing the percentage of representative cell types among all cells from tumor and normal tissues. The two‐tailed Wilcoxon‐ranked *p* value is indicated.

To comprehensively catalog the complexity and dynamic change of diverse compositions of CAFs, we integrated public scRNA‐seq data of human PDAC samples from five studies, including 57 treatment‐naive PDAC tissues, 16 normal pancreas tissues, 5 liver metastatic tissues, and 1 omentum metastatic tissue^[^ ^18^, ^21^, ^22^, ^23^, ^24^ ^]^ (**Figures** **1**A and S1A, Supporting Information). After quality control, 146 185 single cells were retained for subsequent downstream analysis, with 2420 genes and 10881 unique molecular identifiers (UMIs) detected in average each cell. Unsupervised clustering and uniform manifold approximation and projection (UMAP) visualization analysis identified 18 main cell clusters (Figure 1B; Table S1, Supporting Information). The expression pattern of well‐known lineage markers were cross‐referenced for cell type determination (Figure 1C). UMAP plot verified the heterogeneity among multiple cell types rather than background from different studies or tissue sources, which was further validated by highly consistent annotations of cell populations in previous report^[^ ^21^ ^]^ (Figure S1B–D, Supporting Information).

The cell types in the PDAC‐TME exhibited high heterogeneity among different specimens. On a global scale, the majority of cellular compositions were immune cells and ductal cells (Figure 1D and Figure S1E, Supporting Information). Type 1 ductal cells highly expressed the well‐known poor prognosis PDAC markers *KRT19* ^[^ ^25^ ^]^ and were enriched in the PDAC‐TME, indicating that they were malignant ductal cells (Figure 1C,D). Human PDAC tissues contain a dominant proportion (21.0%) of myeloid cells, mainly composed of macrophages and a small number of neutrophils and dendritic cells (DCs). Compared with normal tissues, the abundance of DCs was relative higher in the PDAC‐TME (two‐tailed Wilcoxon‐ranked *t*‐test, *p* = 0.02) (Figure 1E). CD4 T cells (not significant) and regulatory T (Treg; *p* = 0.03) cells were detected almost exclusively from tumors, supporting the accumulation of Treg cells during tumorigenesis (Figure 1E and Figure S1F, Supporting Information). Although more CD8 T cells were detected in tumors (not significant), these cells were characterized by significantly higher expression of exhaustion markers (such as *EOMES* and *LAG3*),^[^ ^26^ ^]^ indicating that tumor‐derived T cells had become exhausted during tumorigenesis (Figure S1G, Supporting Information).

The fibroblasts were derived mostly from PDAC samples (two‐tailed Wilcoxon‐ranked *t*‐test, *p* = 0.03), in line with the highly hyperplastic feature of PDAC tissues (Figure 1E). A total of 12116 fibroblasts were identified with the high expression of marker genes *DCN* (decorin), *LUM* (lumican), and *COL1A1* (collagen type I alpha 1 chain), including 11 116 cells from PDAC tissues (CAFs) and 999 cells from normal tissues (they were NFs) (Figure 1B,C). Altogether, the integrated single‐cell analysis depicts a highly immune‐suppressive microenvironment driven by much more fibroblasts, Tregs, macrophages, immune‐suppressive CD4 T cells, and exhausted CD8 T cells in the human PDAC‐TME.

### 2.2 Comprehensive Atlas of CAFs Reveals Subtypes with Different Biological Characteristics

**Figure 2:**
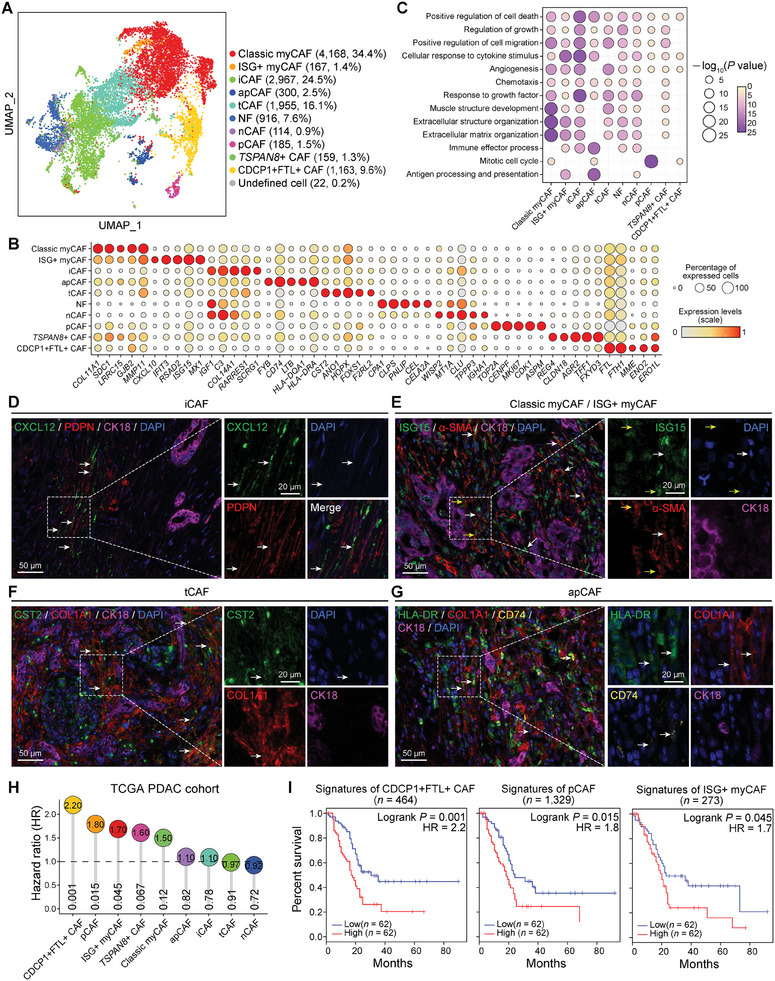
Identification of CAF subtypes. A) UMAP plot showing the clustering of fibroblasts collected from normal or tumor tissues. The dot color indicates CAF subtypes. The number and percentage of cells in each subtype are indicated in brackets. B) Dot plot showing the expression level of top‐ranked five DEGs (ranked by the fold change) in each CAF subtype. Values are scaled in each gene with 0–1 normalization. The dot size indicates the percentage of expressed cells, and the dot color indicates the scaled gene expression level. C) Dot plot showing GO terms of DEGs in each subtype. The dot size and color indicate the statistical significance of the corresponding GO term. D–G) mIF staining showing major CAF subtypes existing in human PDAC tissues. Scale bars, 50 or 20 µm. H) Dot plot showing the HR of distinct CAF subtypes in patients with PDAC from TCGA database (*n* = 124). The value in dots indicates HR and the value in gray bars indicates Logrank *p* value. I) Kaplan–Meier plots showing worse clinical outcome in PDAC patients with the higher expression level of CDCP1^+^FTL^+^ CAFs, pCAFs, or ISG^+^ myCAFs signatures from TCGA database (*n* = 124). +, censored observation; HR, hazard ratio. Logrank *p* values, the number of cell‐type specific marker genes and the number of cases in high/low expression group are indicated.

The fibroblast population could be further divided into 10 subtypes with distinct transcriptional signatures (**Figures** **2**A,B, and S2A–E; Table S1, Supporting Information). All these subtypes expressed pan‐fibroblast markers, such as *COL1A1*, *COL1A2*, *DCN*, and *PDPN*, and these markers were universally present in the PDAC‐TME of different scRNA‐seq cohorts (Figure S2B, Supporting Information). We performed differentially expressed genes (DEGs) to determine transcriptional signatures (cell‐type specific marker genes) and Gene Ontology (GO) analysis to identify biological characteristics of these subtypes (Figure 2C and Figure S2F; Table S2, Supporting Information). The myCAFs and iCAFs were the two main subtypes, accounting for 60.3% of total CAFs (Figure 2A). The myCAFs (4335 cells) showed high expression of *ACTA2*, *TAGLN*, *MMP11*, *MYL9*, *TPM1*/*2/4*, *POSTN*, *IGFBP3*, *THY1*, *COL12A1*, and *THBS2*, further classified into classic myCAFs and interferon simulated genes (ISG)^+^ myCAFs (Figure 2A–C and Figures S2E,F, Supporting Information). Classic myCAFs exhibited high activity of *TGFBI*, *TGFB1I1*, *SMAD2*, and *TWIST1*, as well as the characteristic molecule *PDFGRL* and several myosin proteins of classic myCAFs. ISG^+^ myCAFs were characterized by high expression of almost all known ISGs (for example, *ISG15* and *IFIT1*), as well as genes involved in cytokine‐mediated signaling and inflammatory response pathway (Figure 2B,C). GO analysis revealed that classic myCAFs upregulated specific genes involved in muscle structure development and extracellular structure/matrix organization (Figure 2C).

The iCAFs (24.5% of total CAFs) exhibited enriched expression of chemokines (such as *CXCL2*, *CXCL12*, and *CCL2*), complement components (such as *C3*, *C7*, and *CFH*), and some transcription factor activator protein‐1 (AP‐1) members (such as *FOS*, *JUNB*, and *JUND*) (Figure S2F, Supporting Information). Consistent with previous report, iCAFs also showed an upregulation of *FGF7* ^[^ ^27^ ^]^ (Figure S2F, Supporting Information). iCAFs upregulated genes involved in inflammatory response (such as *A2* *M*, *NFKBIA*, and *FOS*), JAK‐STAT signaling (such as *EGFR*, *IFNGR1*, *MYC*, *PDGFRA*, *STAT3*, and *STAT6*), and cytokine‐mediated pathway (such as *MYC* and *IFITM1*) (Figure 2C). The tCAFs subset displayed biological characteristics between classic myCAFs and iCAFs, suggesting that they may be a group of transitional cells during CAF differentiation.

Due to the rarity and low abundance in the PDAC‐TME, apCAFs were not easily identified and separated from other CAFs in previous reports.^[^ ^21^, ^23^, ^27^ ^]^ However, taking advantage of integrated scRNA‐seq data, the apCAF subtype could be clearly observed, comprising 2.5% of total CAFs. Unlike iCAFs and myCAFs, apCAFs expressed high levels of *CD74* and human leukocyte antigen (HLA) genes (*HLA‐DRA*, *HLA‐DRB1*, *HLA‐DPA1*, and *HLA‐DQB1*) (Figure 2B,C and Figure S2F, Supporting Information). Other upregulated pathways in apCAFs include lymphocyte activation, immune effector process, MTORC signaling, and antigen processing and presentation of peptide antigen via major histocompatibility complex (MHC) class II (Figure 2C). We have confirmed the diverse existence of the aforementioned CAF subtypes using multiplex immunofluorescence (mIF) staining (Figure 2D–G).

Notably, we identified a unique subtype of CDCP1^+^FTL^+^ CAFs that over‐expressed *FTL* and its paralog *FTH1*, and *CDCP1* (Figures S2F,S3A,B, Supporting Information). *FTL* and *FTH1* encode the light and heavy subunit of ferritin protein, respectively.^[^ ^28^ ^]^ These cells showed enriched expression of crucial glycolytic enzymes *ENO1* and *PGK1* (Figure 2B and Figure S2F, Supporting Information). High‐dimensional analysis also identified a new subtype of CAFs with high expresson of membrane protein tetraspanin 8 (*TSPAN8*, hence named as TSPAN8^+^ CAFs) (Figure 2B,C and Figure S2F, Supporting Information), showing enriched expression of sodium/potassium‐transporting ATPase subunit *FXYD3*, *TFF1* and its paralog *TFF3* (Figure 2B and Figure S2F, Supporting Information). In addition, a small subtype of pCAFs was identified, exhibiting co‐expression proliferation related genes such as *MKI67* and *TOP2A* (Figure 2B,C and Figure S2F, Supporting Information).

To examine the clinical implications of diverse CAF subtypes, we conducted survival analysis according to cell‐type specific marker genes in the PDAC cohort from The Cancer Genome Atlas (TCGA) datasets. The analysis revealed that CDCP1^+^FTL^+^ CAFs, pCAFs, and ISG^+^ myCAFs were associated with poor clinical outcomes [hazard ratio (HR) = 2.2, Logrank *p* = 0.001; HR = 1.8, Logrank *p* = 0.015; HR = 1.7, Logrank *p* = 0.045, respectively] (Figure 2H,I). These findings suggested that these CAF subtypes significantly contributed to tumor progression, potentially making them viable therapeutic targets in the future. Overall, these data suggested that CAFs in the PDAC‐TME show highly phenotypic and functional heterogeneity.

### 2.3 CDCP1^+^FTL^+^ CAFs Induced by Hypoxia

**Figure 3:**
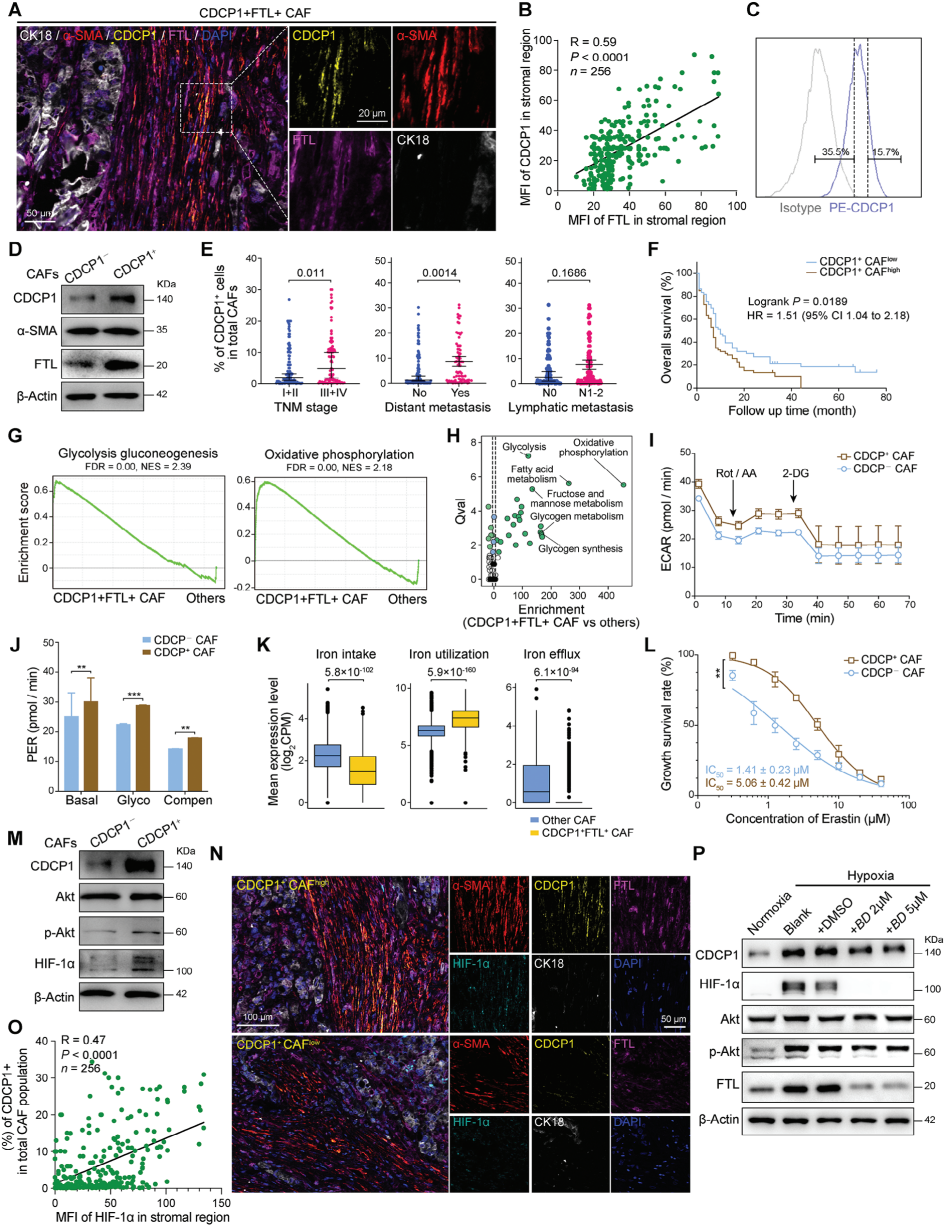
Identification and functional role of CDCP1^+^FTL^+^ CAFs in PDAC. A) Representative images of CDCP1, α‐SMA, FTL, and CK18 immunofluorescence staining in sections of PDAC samples. Scale bars, 50 or 20 µm. B) Scatter plot showing the correlation between the MFI of CDCP1 and FTL in PDAC stromal regions (*n* = 256). The correlation coefficient (*R*) and corresponding test *p* value (*p*) are indicated. C) Representative flow cytometry plot of gating strategy to identify the CDCP1^+/−^ CAF subsets from human primary CAFs (*n* = 3). D) Western blotting showing the CDCP1, α‐SMA, and FTL expression of sorted CDCP1^+/−^ CAFs. E) Scatter plots showing the correlation between the infiltration numbers of CDCP1^+^FTL^+^ CAFs and clinical prognostic factors in PDAC samples (*n* = 196). Data are shown as mean ± SD. Two‐tailed Student's *t*‐test *p* values are indicated. F) Kaplan–Meier survival curves for PDAC patients (*n* = 196) with the low and high infiltration number of CDCP1^+^FTL^+^ CAFs. The median value is the cutoff. Logrank *p* value is indicated. G) GSEA results showing the enriched Kyoto Encyclopedia of Genes and Genomes (KEGG) pathways of CDCP1^+^FTL^+^ CAFs compared with other CAFs. False discovery rate (FDR) and normalized enrichment score (NES) are indicated in the below. H) Scatter plot showing the enrichment level of metabolic pathways in CDCP1^+^FTL^+^ CAFs compared with other CAFs. Representative pathways are indicated. SCPA is performed for this result. I) Representative traces showing real‐time changes in the ECAR in sorted CDCP1^+/−^ CAFs. J) Bar plot showing the quantification of basal glycolysis, the basal proton efflux rate, and compensatory glycolysis in sorted CDCP1^+/−^ CAFs. For each group, *n* = 3 biological replicates. Data are shown as mean ± SD. Two‐tailed Student's *t*‐test *p* values are calculated. ^*^ *p* < 0.05, ^**^ *p* < 0.01, and ^***^ *p* < 0.001. K) Boxplots showing the expression level of iron metabolic pathways. Two‐tailed Student's *t*‐test *p* values are indicated. L) Sorted CDCP1^+/−^ CAFs were treated with the indicated concentrations of Erastin for 48 h, and cell viability was measured by CellTiter‐Lumi. For each group, *n* = 6 biological replicates. Data are shown with the mean value ± SD. *p* value by two‐tailed Student's *t*‐test (paired), ^**^ *p* < 0.01. M) Western blotting showing the CDCP1, Akt/p‐Akt, and HIF‐1α expression of sorted CDCP1^+/−^ CAFs. N) Representative images of CDCP1, α‐SMA, FTL, HIF‐1α, and CK18 immunostaining in sections of PDAC samples. Scale bars, 100 or 50 µm. O) Scatter plot showing the correlation between the percentage of CDCP1^+^ CAFs and the MFI of HIF‐1α in PDAC tissue microarrays (*n* = 256). The correlation coefficient (*R*) and corresponding test *p* value (*p*) are indicated. P) Western blotting showing the CDCP1, Akt/p‐Akt, and HIF‐1α expression of primary CAFs cultured under hypoxia (1% O_2_) with Bruceine D (BD) treatment for 18 h or not.

We performed in situ mIF staining using antibodies against cytokeratin‐18 (CK18), αSMA, FTL, and CDCP1. The analysis confirmed the presence of CDCP1^+^FTL^+^ CAFs in PDAC‐TME (**Figure** **3**A). A significant positive correlation was observed between the mean fluorescence intensity (MFI) of CDCP1 and FTL in the PDAC stromal regions (Figure 3B). Flow cytometry analysis showed that CDCP1 marked a portion of primary CAFs (Figure 3C). The protein expression levels of CDCP1 and FTL in sorted CDCP1^+/−^ CAFs were further validated by Western blotting (Figure 3D). The abundance of CDCP1^+^FTL^+^ CAFs was associated with multiple adverse prognostic parameters, such as advanced stage, distant metastasis, and poor prognosis (Figure 3E,F).

Gene set enrichment analysis (GSEA) and single‐cell pathway analysis (SCPA) revealed the unique metabolic characteristics of CDCP1^+^FTL^+^ CAFs, manifested by enhanced glycolysis and oxidative phosphorylation pathways (Figure 3G,H). To verify the glycolytic activity in CDCP1^+^FTL^+^ CAFs, the extracellular acidification rate (ECAR) was examined in isolated CDCP1^+/−^ CAFs. Compared with CDCP1^‐^ CAFs, increased level of glycolytic ECAR was detected in CDCP1^+^ CAFs (Figure 3I,J).

FTL/FTH is a pivotal iron‐storing nanocage that stores redox‐inactive iron and harbors ferroxidase activity.^[^ ^29^ ^]^ Studies have emphasized that the upregulation of FTH/FTL expression protects cells from ferroptosis.^[^ ^30^, ^31^ ^]^ We compared the expression of ferroptosis‐related metabolic pathways in CDCP1^+^FTL^+^ CAFs and other CAF subsets. The results showed that CDCP1^+^FTL^+^ CAFs exhibited unique iron metabolism, manifested by upregulation of iron utilization and downregulation of iron intake and efflux pathways (Figure 3K and Figure S3C, Supporting Information). The solute carrier family 7 member 11 (SLC7A11), which protects cells from ferroptosis, was significantly upregulated in CDCP1^+^FTL^+^ CAF^[^ ^32^ ^]^ (Figure S3D, Supporting Information). Acyl‐CoA synthetase long‐chain family member 4 (ACSL4), promoting ferroptosis by enriching the cell membrane with long polyunsaturated omega‐6 fatty acids, showed an opposite expression trend^[^ ^33^ ^]^ (Figure S3D, Supporting Information). The mRNA expression levels of *SLC7A11* and *ACSL4* were validated in sorted CDCP1^+^ CAFs and CDCP1^−^ CAFs by real‐time qPCR (RT‐qPCR) (Figure S3E, Supporting Information). When treated with ferroptosis inducer erastin, the reactivity of sorted CDCP1^+^ CAFs was significantly reduced compared to sorted CDCP1^−^ CAFs (Figure 3L).

Considering that hypoxia‐inducible factor (HIF)‐1α drives the resistance of tumor cells to ferroptosis^[^ ^34^ ^]^ and CDCP1 is a downstream target gene of the HIF‐1/2 pathway,^[^ ^35^ ^]^ we speculate that the hypoxic TME may be associated with CDCP1^+^FTL^+^ CAFs. Our analysis showed that the expression of hypoxia related signaling pathway markers, such as HIF‐1α and phosphorylated Akt, were upregulated in sorted CDCP1^+^ CAFs (Figure 3M). Spatial analysis of PDAC tissues revealed that CDCP1^+^FTL^+^ CAFs were enriched in HIF‐1α high expression regions (Figure 3N). A positive correlation was observed between the proportion of CDCP1^+^FTL^+^ CAFs and the intensity of HIF‐1α expression (Figure 3O). Cultivation under hypoxic conditions resulted in enrichment of CDCP1^+^FTL^+^ CAFs, while treatment with HIF‐1α inhibitor Brucaine D reversed the upregulation of CDCP1 and FTL (Figure 3P). In summary, we identified a subpopulation of CDCP1^+^FTL^+^ CAFs driven by hypoxia, exhibiting enhanced glycolysis and resistance to ferroptosis.

### 2.4 apCAFs Exhibit Co‐Expression of Different Immune Cell Signatures

**Figure 4:**
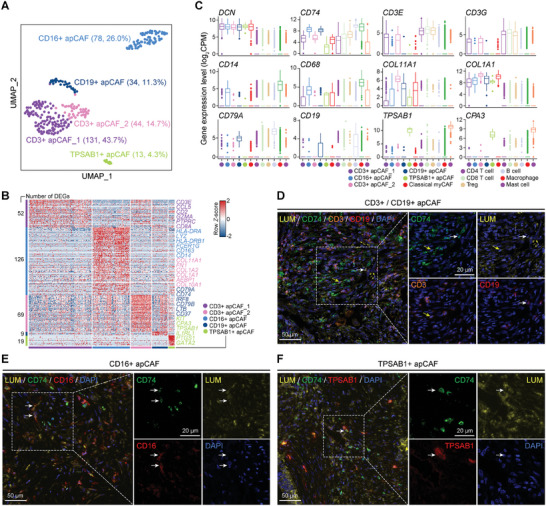
Identification of apCAF subtypes. A) UMAP plot showing the clustering of apCAFs. The dot color indicates apCAF subtypes. The number of cells in each subtype is indicated in brackets. B) Heatmap showing the expression level of DEGs in each apCAF subtype. Values are scaled in each gene with *Z*‐score. The number of DEGs in each subtype is indicated in the left, and representative DEGs are indicated in the right. C) Boxplots showing the expression level of pan‐CAF, apCAF, and immune cell marker genes. Classic myCAFs are used as control. D–F) Imaging of CD3^+^/CD19^+^/CD16^+^/TPSAB1^+^ apCAF in human PDAC tissues using mIF. Scale bars, 50 or 20 µm.

In comparison to other CAFs, apCAFs exhibited specific overexpression of MHC‐II molecules (*HLA‐DP/DQ/DR*), while displaying similar levels of MHC‐I molecules (*HLA‐A/B/C/F/G*) (Figure S4A, Supporting Information). We found that apCAFs have high heterogeneity and could be divided into five subtypes, co‐expressing marker genes of apCAFs (including *DCN* and *CD74*) and different immune cell lineage markers (**Figures** **4**A,B and S4B,C, Supporting Information). The subtypes of CD3^+^ apCAF_1 and CD3^+^ apCAF_2 specifically expressed T‐cell marker genes *CD3E* and *CD3G*, CD19^+^ apCAFs specifically expressed B cell marker genes *CD19* and *CD79A*, CD14^+^ apCAFs specifically expressed macrophage marker genes *CD14* and *CD68*, and TPSAB1^+^ apCAFs specifically expressed mast cell marker genes *TPSAB1* and *CPA3* (Figure 4A–C). Using mIF staining, we confirmed the presence of these apCAF subtypes in human PDAC samples (Figure 4D–F). These results suggested that apCAFs are a highly heterogeneous population. In addition to participating in T‐cell antigen presentation, apCAFs may also interact with B cells, macrophages, and mast cells, leading to the shaping of different phenotypes. The underlying molecular mechanism needs further in‐depth study.

### 2.5 Evolution and Potential Differentiation Trajectories of Different CAF Subtypes

**Figure 5:**
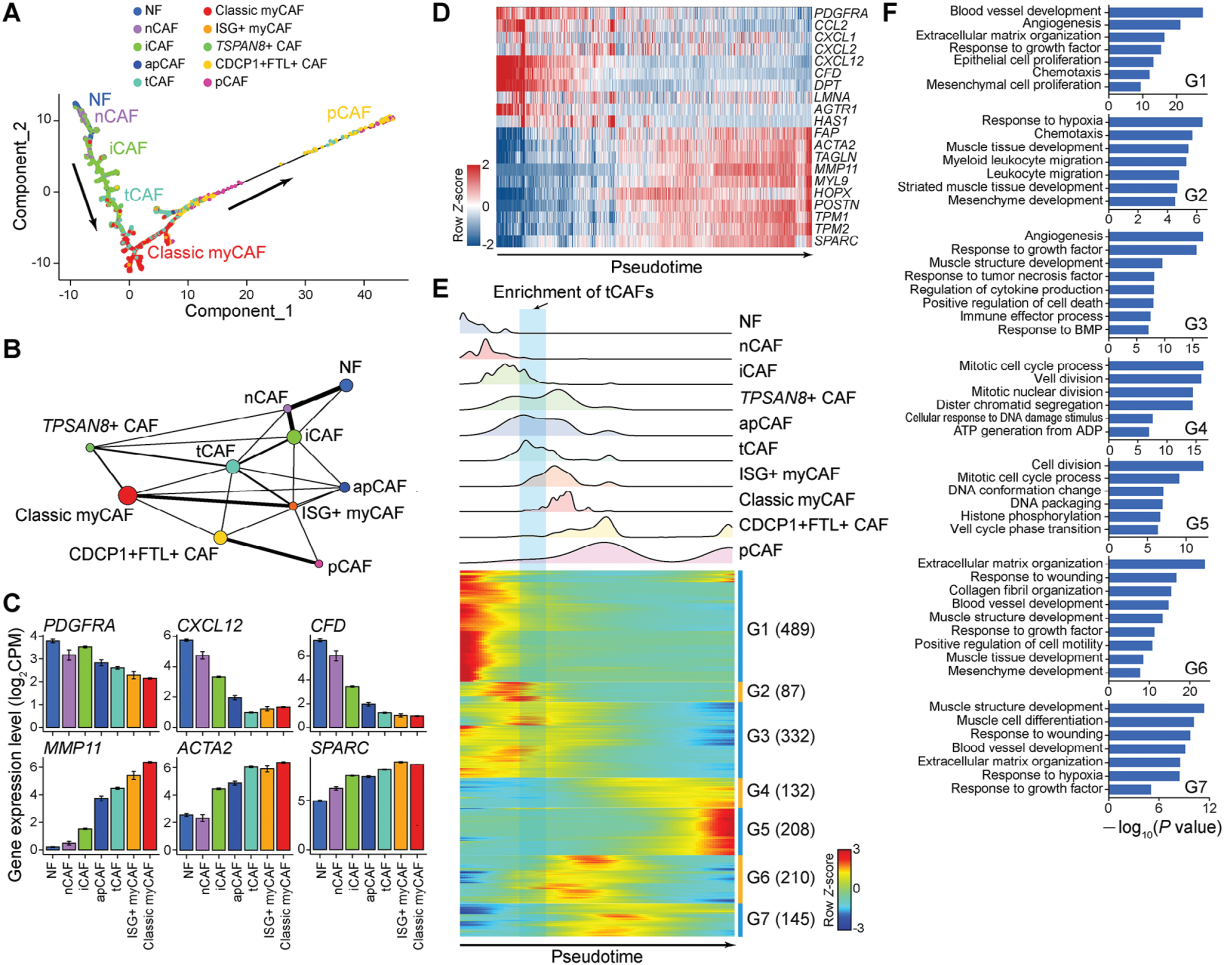
Identification of differentiation trajectory of diverse CAF subtypes. A) Pseudotime analysis showing the differentiated trajectory of distinct CAF subtypes. The dot color indicates cell types. The arrow indicates the pseudotime from early to late time. B) PAGA result showing the transcriptome similarity among CAF subtypes. The dot color and size indicate the cell type and cell number, respectively. C) Bar plots showing the expression level of iCAF (top panel) and myCAF (bottom panel) marker genes in CAF subtypes. Data are shown with the mean value ± SD. D) Heatmap showing the expression level of representative iCAF and myCAF marker genes along pseudotime. Values are scaled in each gene with *Z*‐score. E) Top, density plot showing the number distribution of cells in distinct CAF subtypes along pseudotime. Bottom, heatmap showing the expression level of dynamic genes along pseudotime. Values are scaled in each gene with *Z*‐score. The number of dynamic genes in each group (from G1 to G7) is indicated in brackets. The blue shadow indicates the pseudotime interval in which tCAFs are enriched. F) Bar plots showing GO terms of dynamic genes in each group (from G1 to G7), corresponding to Figure 5E.

The CAF subtypes exhibit differentiate plasticity and reversible cell states under specific in vitro culture conditions, but the differentiation trajectory of these CAF subtypes is still unclear.^[^ ^18^, ^20^ ^]^ Cell lineage pseudotime inference analysis showed a gradually transition from NFs to nCAFs, iCAFs, and apCAFs, to ISG^+^ and classic myCAFs, and eventually to pCAFs (**Figure** **5**A and Figure S5A,B, Supporting Information). Partition‐based graph abstraction (PAGA) analysis confirmed the potential differentiation trajectory of diverse CAF subtypes (Figure 5B).^[^ ^36^ ^]^ We found a shared pattern of transcription between iCAFs and NFs/nCAFs, suggesting that iCAFs may directly originate from nCAFs (Figure 5A,B and Figure S2E,F, Supporting Information). NFs and nCAFs highly expressed *CFD*, *PDGFRA*, and *CXCL12*, which have been identified as iCAF‐related genes (Figure 2B). The expression level of these genes gradually decreased along the trajectory leading to apCAFs, tCAFs, ISG^+^ myCAFs, and classic myCAFs (Figure 5C,D). In parallel, the gradual increase in the expression level was observed for signature genes of classic myCAFs (for example, *MMP11*, *ACTA2*, and *SPARC*) along the trajectory, revealing a spectrum of phenotypic states (Figure 5C,D). For example, *ACTA2* and *PDGFRA* displayed an inverse relationship across classic myCAFs and iCAFs, respectively. This expression pattern was consistent with a one‐dimensional continuum curve formed by iCAFs and classic myCAFs, suggesting a gradual shift and reversible cell states between iCAFs and classic myCAFs.

To further investigate the molecular characteristics along diverse CAF subtypes transition, we identified genes whose expression level changed along with the pseudotime. The obtained genes were clustered into seven groups from G1 to G7 based on expression profiles, and corresponding GO analysis was performed (Figure 5E,F; Table S2, Supporting Information). The tCAFs highly expressed genes of G2 and G3 which were enriched in the muscle development, leukocyte migration and immune effector process (Figure 5E,F). Consistently, compared with iCAFs and classic myCAFs, tCAFs exhibited the intermediate expression level of markers genes of iCAFs and classic myCAFs, and tCAFs showed a continuum of cell states bounded by these two major transcriptional programs (Figure 5C). We also noticed that tCAFs link iCAFs and classic myCAFs in the UMAP plot (Figure 2A). In summary, we inferred a differentiation trajectory of diverse CAF subtypes originating from NFs, with tCAFs representing an intermediate state in the gradual shift between iCAFs and classic myCAFs.

### 2.6 AP‐1 Family Triggers Malignant Transformation of NFs

**Figure 6:**
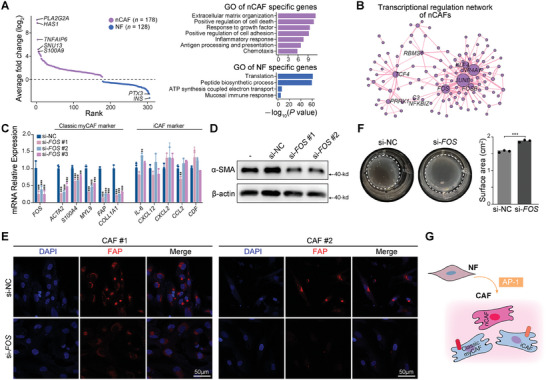
AP‐1 members triggering the malignant phenotype conversion of NFs. A) Left, scatter plot showing the DEGs between nCAFs and NFs. The pink dot indicates the highly expressed DEGs (*n* = 178) in nCAFs, and the purple dot indicates the highly expressed DEGs (*n* = 128) in NFs. The representative DEG is indicated. Right, barplots showing GO terms of nCAF (top) or NF (bottom) specific genes. B) Transcriptional regulation network showing potential core regulators in nCAFs. top‐ranked regulators (ranked by the number of connections) are indicated. The dot size indicates the number of connected genes, and the line thickness indicates the weight of gene pairs. Only connections with high weights are conserved. C) Bar plots showing the mRNA expression of representative genes of classic myCAF and iCAF in human primary CAFs after knocking‐down *FOS* by si‐RNA. si‐*FOS1* #1/2/3 represent three different siRNAs targeting *FOS* for avoiding off‐target effect. For each group, *n* = 3 biological replicates. Data are shown with the mean value ± SD. *p* value by one‐way ANOVA, ^*^ *p* < 0.05, ^**^ *p* < 0.01, ^***^ *p* < 0.001, ^****^ *p* < 0.0001. D) Representative Western blot showing α‐SMA levels in human primary CAFs after knocking‐down *FOS* by si‐RNA. E) Representative of confocal microscopy images of FAP staining in human PDAC CAFs after knocking‐down *FOS* by si‐RNA. CAF#1/2 represent different two lines of patient‐derived CAFs. Scale bar, 50 µm. F) Left, the gel contractile ability of human PDAC CAF transfected with si‐*FOS*. Right, barplot showing the quantified gel contractile ability. For each group, *n* = 3 biological replicates. Two‐tailed Student's *t*‐test *p* value is calculated. Data are shown as mean ± SD. ^***^ *p* < 0.001. G) Cartoon depicting AP‐1 members triggering the malignant phenotype conversion of NFs.

Different origins of CAFs have been proposed, including tissue‐resident fibroblasts,^[^ ^37^ ^]^ bone‐marrow derived mesenchymal stem cells,^[^ ^38^, ^39^ ^]^ and adipocytes.^[^ ^40^, ^41^ ^]^ Hierarchical clustering, differentiation trajectory analysis, and correlation analysis all supported the transcriptional similarity between NFs and nCAFs, indicating a potential transition from NFs to nCAFs (Figure 5A,B). To explore the molecular cues inducing the transformation of nCAFs, we identified 178 upregulated and 128 downregulated DEGs in nCAFs compared to NFs (**Figure** **6**A; Table S2). GO analysis revealed that upregulated DEGs in nCAFs were involved in epithelial mesenchymal transition (EMT; for example, *SERPINH1*, *VCAN*, *SPARC*, and *POSTN*), ECM organization (for example, *COL1A1*, *COL3A1*, *MMP7*, and *MMP14*), inflammatory response (for example, *C3*, *S100A9*, *TNFAIP6*, and *CXCR4*), as well as antigen processing and presentation of peptide antigen through MHC class II (for example, *CD74*, *HLA‐DRA*, and *HLA‐DRB1*) (Figure 6A and Figure S6A, Supporting Information). The costimulatory molecules *CD276* (B7‐H3) and *TNFSF10* (TNF superfamily member 10) were significantly expressed in nCAFs (Figure S6A, Supporting Information). On the contrary, compared with nCAFs, *CLPS* (pancreatic colipase preproprotein), *CFD* (complement factor D), ATP synthesis and oxidative phosphorylation‐related genes (for example, *COX5B*, *COX6C*, *COX7C*, and *NDUFA4*), as well as metabolism‐related genes (for example, *ALDH1A1*, *CEL*, *GLUL*, *LGALS1*, and *PRDX6*) were highly expressed in NFs (Figure 6A and Figure S6A, Supporting Information).

Transcriptional regulation network analysis revealed upregulation of several pivotal transcription regulators in nCAFs, including AP‐1 family members (*FOS*, *FOSB*, and *JUNB*) and *NR4A1* (Figure 6B). Previous studies have revealed that activated FOS and JUN families are necessary and sufficient for maintaining CAF‐specific enhancers activation in cancers.^[^ ^42^, ^43^ ^]^ Therefore, we assumed that the AP‐1 family played a crucial role in inducing the phenotype transition of CAFs. To verify the regulatory role of the AP‐1 family, we isolated paired CAFs and NFs from human PDAC tissues. RT‐qPCR and flow cytometry confirmed their fibroblast characteristics, showing over‐expression of mesenchymal marker vimentin, but lacking expression of epithelial (EPCAM), endothelial (CD31), or immune (CD45) markers (Figure S6B,C, Supporting Information). When isolated primary CAFs were cultured in vitro, they exhibit a myofibroblast‐like phenotype and produce high levels of FAP and αSMA compared to matched NFs (Figure S6D,E, Supporting Information). Then, we knock‐downed the expression of *FOS*, *FOSB*, *JUN*, and *NR4A1* in CAFs using specific si‐RNAs. The results showed that *FOS* knockdown led to a significant downregulation of the mRNA levels of classic myCAF markers, while *JUN* knockdown led to a significant downregulation of iCAF markers (Figure 6C and Figure S6F, Supporting Information). Knockdown of *FOSB* or *NR4A1* showed a mild effect on the expression of CAF markers, but no statistically significant difference was observed (Figure S6F, Supporting Information). Consistently, knockdown of *FOS* in CAFs significantly reduced the expression of classic myCAF markers FAP and α‐SMA at the protein level (Figure 6D,E), and inhibited the gel contraction ability of CAFs (Figure 6F). Taken together, these results revealed that AP‐1 family members play an important role in the phenotypic transformation of CAF, thereby promoting tumor progression (Figure 6G).

### 2.7 IFNγ Promotes the Reverse Transformation from Classic myCAFs into iCAFs

**Figure 7:**
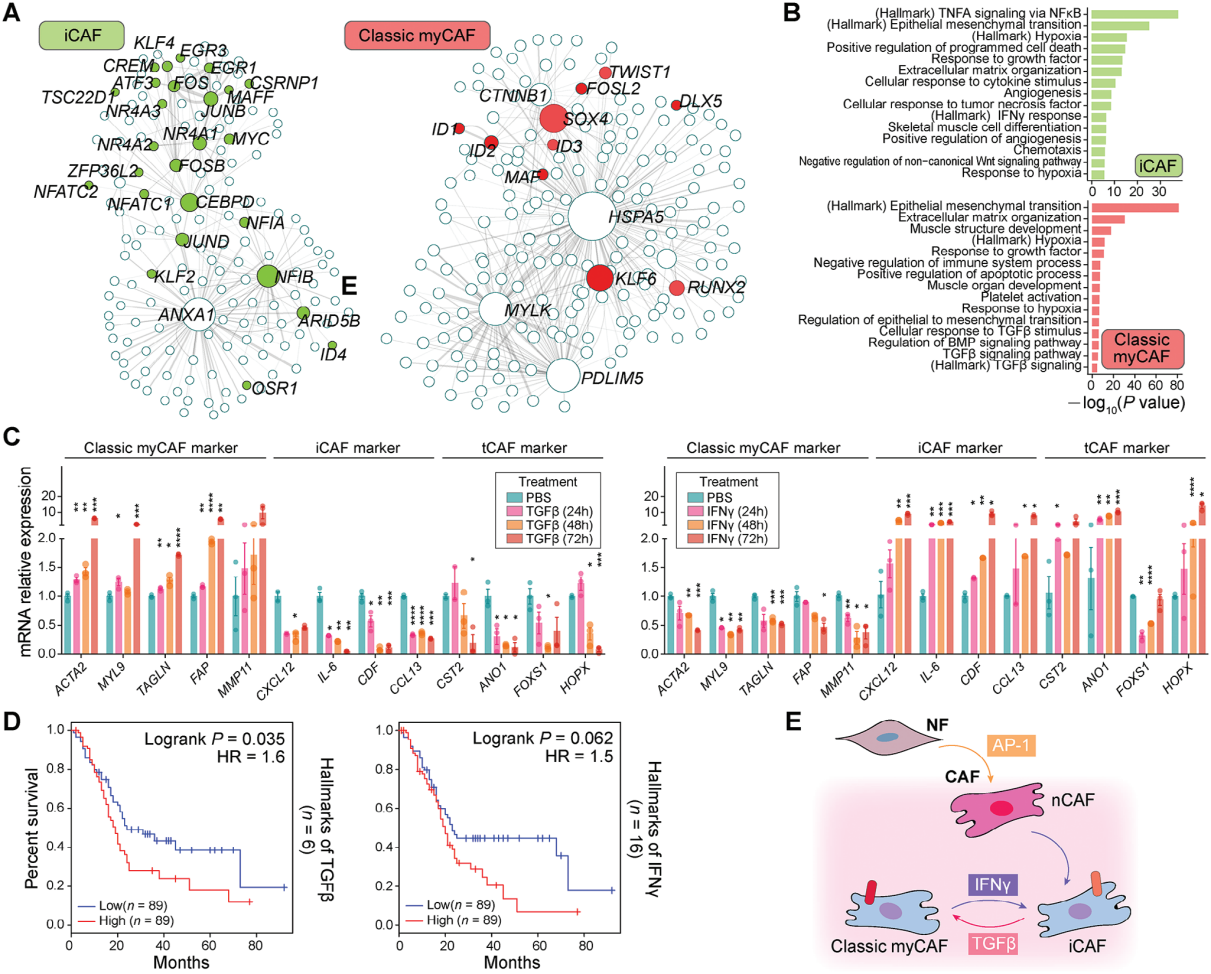
IFNγ contributing to the fate transformation from classic myCAFs to iCAFs. A) Transcriptional regulation networks showing potential core regulators in iCAFs (left) and classic myCAFs (right). Regulators existing in the dynamic gene set along pseudotime (corresponding to Figure 5E) are indicated in green (left) or red (right), and names of these regulators and top‐ranked genes (ranked by the number of connections) are indicated. The dot size indicates the number of connected genes, and the line thickness indicates the weight of gene pairs. Only connections with high weights are conserved. B) Bar plots showing GO terms of genes in transcriptional regulation networks, corresponding to Figure 7A. C) Bar plots showing the mRNA expression of representative genes of classic myCAF, iCAF, and tCAF in human primary CAFs after treating with TGFβ (20 ng/mL; left) or IFNγ (20 ng/mL; right) at 24, 48, and 72 h. For each group, *n* = 3 biological replicates. Data are shown with the mean value ± SD. *p* value by one‐way ANOVA, ^*^ *p* < 0.05, ^**^ *p* < 0.01, ^***^ *p* < 0.001, ^****^ *p* < 0.0001. D) Kaplan–Meier plot showing better clinical outcome in PDAC patients with the lower expression level of TGFβ (top) or IFNγ (bottom) hallmarks. +, censored observation; HR, hazard ratio. Logrank *p* values, the number of signature genes (at the right of each panel) and the number of cases in high/low expression group are indicated. E) Cartoon depicting IFNγ contributing to the fate transformation from classic myCAFs to iCAFs.

In the scRNA‐seq dataset, classic myCAFs and iCAFs are the main CAF subtypes (account for 58.8% of all). It has been widely reported that classic myCAFs and iCAFs displayed reversible cell states,^[^ ^20^ ^]^ but the potential regulators and driving factors remain to be explored. To investigate the potential fate transition between these two CAF subtypes, we identified a range of potential core regulatory factors whose expression levels are dynamic along the pseudotime and serve as cell‐type specific markers, based on the above differentiation trajectory analysis. Transcriptional regulatory networks of iCAFs and classic myCAFs were separately established (**Figure** **7**A).

Consistent with the indicated activation of TGFβ/BMP signaling pathways in classic myCAFs, transcription regulators (such as *SOX4*, *TWIST1*, *ID1*, *ID2*, *ID3*, *RUNX2*, and *KLF6*), which were known target genes of the TGFβ pathway or were reported to be induced by TGFβ signaling, were upregulated in classic myCAFs (Figure 7A,B and Figure S7A, Supporting Information).^[^ ^44^, ^45^ ^]^ When we treated CAFs in vitro with TGFβ, RT‐qPCR analysis revealed a significant increase in the expression level of classic myCAF markers and a significant decrease in that of iCAF markers, suggesting the composition shift of iCAFs and classic myCAFs (Figure 7C). It is in line with the previous evidence exhibiting the conversion of iCAFs to myCAFs via the TGFβ activation.^[^ ^20^ ^]^ Furthermore, in the TCGA dataset, the high expression level of TGFβ (log‐rank *p* = 0.035) suggested an unfavorable prognosis in PDAC patients (Figure 7D).

It was proven that IL1 induced leukemia inhibitory factor (LIF) expression and downstream JAK/STAT activation to generate inflammatory CAFs.^[^ ^20^, ^46^ ^]^ However, our scRNA‐seq analysis displayed that IFNγ response‐related genes (for example, *CEBPD*, *NFKBIA*, *TXNIP*, and *MT2A*) also play important roles in the iCAF phenotype maintain and function regulation (Figure 7A,B and Figure S7B, Supporting Information). Therefore, we assumed that IFNγ may be an important proinflammatory molecule driving the conversion of classic myCAFs to iCAFs. To validate this, we treated CAFs in vitro with IFNγ, and detected a significant increase in the expression level of iCAF markers, and a decrease in that of classic myCAF markers, suggesting that IFNγ triggered the conversion of classic myCAFs into iCAFs (Figure 7C). This finding supports that proinflammatory factors IFNγ can induce a transition from classic myCAFs to iCAFs, consistent with that the elevated expression level of IFNγ (log‐rank *p* = 0.062) was correlated with an unfavorable prognosis in PDAC patients (Figure 7D).

As above mentioned, tCAFs expressed the intermediate levels of iCAFs and classic myCAFs markers genes, and exhibited continuity of cellular state restricted by these two main transcriptional programs. To verify the biological characteristics of tCAF linking myCAFs and iCAF transition during the process, we also detected the expression levels of tCAF subpopulation specific marker genes (*CST2*, *ANOX1*, *FOXS1* and *HOPX*) after TGFβ or IFNγ treatment for 24, 48, and 72 h. Interestingly, we also observed dynamic changes in mRNA expression levels of specific marker genes of tCAFs (Figure 7C). The result indicated that tCAFs represent an intermediate state between the iCAF and myCAF phenotypes, further supporting the plasticity between these two CAF populations. In conclusion, our results identified several potential driving factors, including IFNγ and TGFβ, to promote the reversible conversion between classic myCAFs and iCAFs (Figure 7E).

### 2.8 Interaction between CAFs and Myeloid Cells Reveals *CXCL12‐CXCR4* as a Therapeutic Target

**Figure 8:**
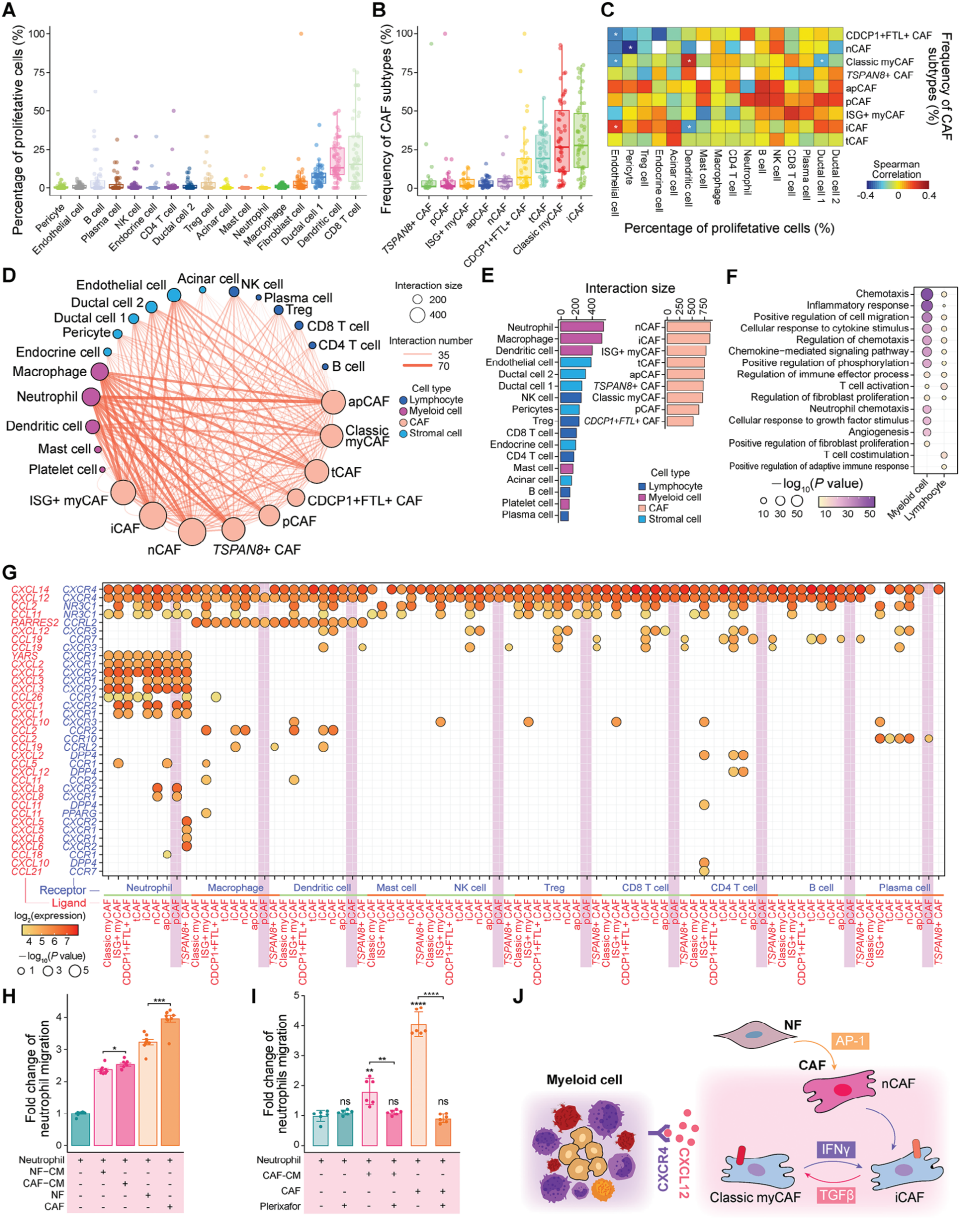
Cell–cell interactions between CAF subtypes and myeloid cells. A) Boxplot showing the percentage of *MKI67* ^+^ cells in each cell type within tumor tissues. Each dot corresponds to each sample. B) Boxplot showing the frequency of each CAF subtype in tumors. Each dot corresponds to each sample. C) Heatmap showing the Spearman correlation coefficient between the abundance of CAF subtypes and the proliferation percent of other cells in tumors. Correlation test *p* values are indicated, ^***^ *p* < 0.001, ^**^ *p* < 0.01, and ^*^ *p* < 0.05. D) Cell–cell interaction network showing interactions between CAF subtypes and other cells in tumors. The dot color indicates the cell type, the dot size indicates the interaction number of a given cell type, and the line thickness indicates the interaction number of a given cell type pair. E) Bar plots showing the interaction number of a given cell type. F) Dot plot showing GO terms of ligands and receptors within specific myeloid cells‐CAFs interactions and specific lymphocytes‐CAFs interactions. G) Dot plot showing the expression level of ligand‐receptor gene pair related with chemotaxis and cytokine within myeloid cell‐CAF interactions. The dot color and size indicate the expression levels and statistical significance, respectively. Red words indicate the ligands (row) expressed in the corresponding cell type (column), and blue words indicate the receptors (row) expressed in the corresponding cell type (column). H) Bar plot showing the fold change of neutrophil migration obtained following the addition of the CAFs/NFs or the CM derived from CAFs/NFs in lower chamber after 1 h. For each group, *n* = 7 biological replicates. Data are shown as mean value ± SD. One‐way ANOVA *p* values are calculated. ^*^ *p* < 0.05 and ^***^ *p* < 0.001. I) Neutrophils were treated with or without CXCL4 inhibitor Plerixafor (25 µM). Neutrophil migration assay was performed by adding control medium or culture medium of CAFs into lower champers, or seeding CAFs into lower champers with or without Plerixafor (25 µM). Bar plot showing the fold change of neutrophil migration after 1 h. For each group, *n* = 7 biological replicates. Data are shown as mean value ± SD. One‐way ANOVA *p* values are calculated. ^**^ *p* < 0.01, ^****^ *p* < 0.0001, and ns, not significant. J) Cartoon depicting *CXCL12‐CXCR4* as a major interaction axis between CAFs and myeloid cells.

To reveal potential regulation relationship across cell types in the PDAC‐TME, we correlated the abundance of CAFs and proliferation fractions of other cells measured by the percentage of *MKI67* ^+^ cells, which may reflect direct regulation function (**Figure** **8**A–C). The abundance of iCAFs were positively correlated with the proliferation of endothelial cells (Spearman correlation coefficient *ρ* = 0.31, correlation test significance *p* = 0.03), while classic myCAFs were anti‐correlated with the proliferation of type 1 ductal cells (*ρ* = −0.29, *p* = 0.04).

For detecting the changed cell–cell interactions and ligand–receptor pairs after tumorigenesis, we analyzed cell–cell interactions in normal and tumor tissues, respectively (Figure 8D and Figure S8A, Supporting Information). Interestingly, compared with normal tissues, much more newborn cell–cell interactions were detected between CAFs and neutrophils, and between CAFs and macrophages during PDAC progression (Figure S8B). Infiltration of myeloid cells negatively correlates with prognosis,^[^ ^47^ ^]^ and numerous interactions between myeloid cells and CAFs were consistent with an immune‐suppressive role of these cells.

We further investigated the CAF subtypes that most frequently interacted with immune or other somatic cells in the PDAC‐TME. Our findings revealed that myeloid cells, particularly neutrophils, were the top‐ranking cell types that interacted most frequently with CAFs (Figure 8D,E). We identified 718 specific interactions (corresponding to 97 specific genes) between myeloid cells and CAFs, in contrast to 188 specific interactions (corresponding to 34 specific genes) between lymphocytes and CAFs. According to GO analysis results, we found that specific ligands and receptors within myeloid cell‐CAF interactions were associated with the chemotaxis, inflammatory response, cell migration, and cell adhesion process, while specific ligands and receptors within lymphocyte‐CAF interactions were involved in T‐cell co‐stimulation and positive regulation of adaptive immune responses (Figure 8F). Of note, different from antigen presenting cells (APCs) expressing costimulatory molecules on cell surface, all CAF subtypes lowly expressed costimulatory genes, including *CD40*, *CD80*, and CD86 (Figure S8C, Supporting Information). It suggested that the interactions between CAFs and other cells were different from professional APCs.

Among myeloid cell‐CAF specific interactions, we detected 25 chemotaxis and cytokine‐related interactions especially in neutrophil‐CAF interactions, including *CXCL12‐CXCR4*, *YARS‐CXCR1*, and *CXCL2*‐*CXCR2* (Figure 8G and Figure S8D, Supporting Information). We noticed that the gene pair *CXCL12‐CXCR4* was commonly expressed within interactions in which CAFs expressed ligand *CXCL12* and other cells expressed receptor *CXCR4* (Figure 8G and Figure S8E, Supporting Information). However, *CXCL12‐CXCR4* was rarely detected within interactions among other cell types except for CAFs, inferring that *CXCL12‐CXCR4* may be a potential key interaction between CAFs and other cells (Figure S8E, Supporting Information). To validate this chemotactic effect, we established co‐culture systems of neutrophils with CAFs and NFs (direct) or their corresponding culture medium (CM) (indirect). Both CAFs and CAFs‐CM exhibited stronger chemotactic effects towards neutrophils compared to NFs and NFs‐CM (Figure 8H). By blocking CXCR4 on neutrophils with Plerixafor, the chemotactic effects of CAFs or CAFs‐CM were eliminated, further demonstrating the CXCR4‐CXCL12 axis mediated interaction between CAFs and neutrophils (Figure 8I).

Altogether, we observed that CAFs frequently interacted with myeloid cells in the PDAC‐TME, and therefore, therapeutic disruption of the crosstalk between CAFs and myeloid cells would be beneficial during treatment (Figure 8J).

## 3 Discussion

Currently, the complete range of functional heterogeneity, differentiation plasticity, and potential mutual transformation among diverse CAF subtypes in the human PDAC‐TME remain incompletely understood. In this study, we endeavored to address these questions using an unbiased approach, independent of prior knowledge, to characterize CAF subtypes and provided their molecular signatures. Our findings introduce the concept of a dynamic PDAC‐TME of CAFs, wherein spatially stable cells adapt their transcriptional program to accommodate the evolving tumor ecosystem.

Leveraging the accumulated scRNA‐seq data encompassing immune cells and somatic cells, we not only unveiled six well‐established CAF subtypes but also identified three novel subtypes within the human PDAC‐TME. In the human PDAC‐TME, myCAFs are spatially located close to tumor cells,^[^ ^20^ ^]^ indicating a direct cell–cell interaction. Furthermore, a separate study revealed that this direct interaction triggers CAFs to express a wide range of inflammatory modulators, a process that stimulates ISG transcriptional programs in CAFs and subsequently impairs the effectiveness of oncolytic viruses.^[^ ^48^ ^]^ Consequently, we postulated the presence of myCAFs exhibiting high ISG expression levels. Utilizing scRNA‐seq data, we distinguished ISG^+^ myCAFs, and observed that ISG^+^ myCAFs exhibited a closer proximity to tumor cells compared to other myCAFs. These findings supported the distinctive functional role of ISG^+^ myCAFs’ inflammatory modulators, and indicated their potential origination from myCAFs after stimulation by tumor cells.

We also identified a distinct subtype of CAFs, characterized by high expression of *FTL* and its paralog *FTH1*, designated as CDCP1^+^FTL^+^ CAFs. CAFs were considered to produce metabolic intermediates as a fuel source for cancer cells to favor PDAC progression.^[^ ^49^ ^]^ CDCP1^+^FTL^+^ CAFs exhibited similar transcriptional profiles to previously characterized meCAFs,^[^ ^50^ ^]^ both of which displayed a highly active glycolysis pathway. Through the surface marker CDCP1, we enabled live cell isolation of this specific subtype and demonstrated their augmented glycolysis. We further demonstrated that PDAC patients with the elevated proportion of CDCP1^+^FTL^+^ CAFs in TME suffered from advanced cancer stage, distant metastasis, and poor outcomes. Our findings underscore the potential of targeting CDCP1^+^FTL^+^ CAFs as a therapeutic strategy to enhance prognosis for PDAC patients. Interestingly, we uncovered an important function of this subtype in regulating ferroptosis, a form of cell death triggered by iron‐dependent phospholipid peroxidation, which presents a potential therapeutic opportunity to impede cancer progression.^[^ ^51^, ^52^ ^]^ It has been reported that CAFs support glutathione synthesis in PDAC through augmenting extracellular cysteine supply, thereby preventing against ferroptosis.^[^ ^53^ ^]^ In our study, CDCP1^+^FTL^+^ CAFs were distinguished by their enhanced iron metabolism and resistance to ferroptosis. Hence, our discoveries emphasized the significance of the functional traits of CDCP1^+^FTL^+^ CAFs when developing therapeutic strategies centered on ferroptosis induction. Furthermore, we identified hypoxia as a key inducer of the CDCP1^+^FTL^+^ CAF phenotype, which could be reversed by inhibiting HIF‐1α. Altogether, our results supported a promising therapeutic strategy that combines hypoxia alleviation with ferroptosis induction, achieved by restricting the differentiation of this specific CAF subpopulation.

The apCAFs were observed in the PDAC‐TME several years ago, with an initial report indicating their ability to activate CD4 T cells.^[^ ^18^ ^]^ However, prior studies have revealed contrasting roles of apCAFs. For instance, those originating from mesothelial cells in the PDAC‐TME contribute to tumor immunosuppression by inducing the formation of regulator T cells,^[^ ^54^ ^]^ while apCAFs derived from ATII cells in non‐small lung cancer actively enhance immune function.^[^ ^55^ ^]^ These findings imply that apCAFs stemming from different sources may exhibit diverse functions, necessitating further investigation. Intriguingly, our research unveiled distinct subtypes of apCAFs, co‐expressing various immune cell markers, including B cells, T cells, macrophages, and mast cells, hinting at the multifaceted immune‐modulatory capacities of apCAFs in conjunction with immune cells. Our results suggested that apCAFs are a highly heterogeneous population. In addition to participating in T‐cell antigen presentation, apCAFs may also interact with other immune cells, leading to the shaping of different phenotypes. The underlying molecular mechanism needs further in‐depth study.

It has been demonstrated that CAF can originate from different sources.^[^ ^37^, ^38^, ^39^, ^40^, ^41^, ^56^ ^]^ Given that NFs and nCAFs share common molecular characteristics, we initially examined the potential molecular drivers responsible for triggering the conversion of tissue‐resident fibroblasts into a malignant phenotype. Our bioinformatics and in vitro experiment results indicated that *JUN* may play a regulatory molecule that promotes the iCAF‐related signatures, while *FOS* upregulate the myCAF‐related features in the malignant transformation of NFs. It has been proven that ANKRD1, a mesenchymal‐specific transcriptional coregulator in human dermal fibroblasts (HFDs), interacts with AP‐1 transcription factors to bind CAF effector genes, ultimately promoting the production of CAFs from HFDs.^[^ ^57^ ^]^ Moreover, it has been demonstrated that *JUN* overexpression in human embryonic lung fibroblasts resulted a significant enrichment of iCAF‐related signatures but not myofibroblastic CAF signature.^[^ ^58^ ^]^ Consistently, these findings revealed that AP‐1 family members collectively play a pivotal role in the malignant phenotype conversion of NFs into CAFs.

Upon further investigation into the evolution and differentiation of distinct CAF subtypes with malignant phenotypes, our findings supported their differentiation plasticity. The previous study has demonstrated that iCAFs can be differentiated from pancreatic stellate cells and PDAC organoid‐conditioned medium co‐cultures through contact‐independent mechanisms, indicating the reversible nature of classic myCAFs and iCAFs in vitro.^[^ ^20^ ^]^ Additionally, apCAFs from mouse PDAC also have the differentiation plasticity, which could change into classic myCAFs in appropriate culture conditions.^[^ ^18^ ^]^ Compared with prior researches, our study provides a more comprehensive differentiation trajectory among CAF subtypes in the human PDAC‐TME, from nCAFs, iCAFs, and apCAFs, to classic and ISG^+^ myCAFs, and eventually to pCAFs. The classic myCAFs and iCAFs emerged as the primary and reversible CAF subtypes, and our investigation delved into the specific master transcription regulators involved in the polarization process of these two CAF subtypes. During the process of CAF polarization, specific master transcription factors are activated and mutually exclusive effector molecules are expressed. We observed that the transcriptional characteristics of polarized CAF subtypes are not entirely mutually exclusive, underscoring the inherent plasticity of distinct CAFs. For instances, genes related to muscle cell differentiation were also expressed in iCAFs. Intriguingly, we newly revealed that the proinflammatory factor IFNγ can induce the conversion of classic myCAFs into iCAFs. Within classic myCAFs, several novel therapeutic strategies targeting ECM deposition have been developed to impair tumor growth.^[^ ^59^ ^]^ Therefore, newly identified driving factors would contribute to block the conversion of classic myCAFs into iCAFs, and CAF‐targeting therapeutic approaches directly target classic myCAFs instead of several CAF subtypes in the future.

In addition to serving as a physical barrier to lymphocyte infiltration, our cell–cell interaction analysis unveiled the recruitment function of CAFs to myeloid cells, particularly neutrophils and macrophages, which are both associated with a poor clinical prognosis due to their immunosuppressive properties and roles in mediating therapeutic resistance.^[^ ^60^, ^61^, ^62^ ^]^ Similar to the mechanism by which neutrophils attract tumor cells,^[^ ^60^, ^63^ ^]^ our findings indicate that CAFs recruit neutrophils through chemotaxis, exemplified by the *CXCL12‐CXCR4* Interaction. The suppression of neutrophils by lorlatinib has been shown to attenuate pancreatic cancer growth and improve treatment with immune checkpoint blockade.^[^ ^64^ ^]^ Our result underscores the importance of targeting the CAF‐neutrophil axis to enhance cancer treatment.

In summary, our study offers a comprehensive characterization of CAFs in the human PDAC‐TME, encompassing the definition of CAF subtypes, the elucidation of differentiation trajectory or plasticity, and the observation of interactions between CAFs and myeloid cells. We provide valuable insights into the potential roles of CAFs in modulating the immune microenvironment and identify potential therapeutic targets for cancer treatment.

## 4 Experimental Section

### Collection of Patients and Tissue Samples

Experiments with human tissues were authorized by the Human Ethics Committee of Shanghai General Hospital, Shanghai Jiao Tong University School of Medicine (Shanghai, China) with Institutional Review Board approval ([2017] 53) and all patients were consent to participate in this study.

### Integrating scRNA‐seq Data

Since the batch effect induced by experiment or other factors in different studies, scRNA‐seq data were firstly integrated with R package *Seurat*.^[^ ^65^ ^]^ In brief, only genes expressed in at least five cells were considered and cells with gene number greater than 500 were retained as high‐qualify cells in the downstream analysis. Each dataset was individually normalized using function *NormalizeData* with log‐transform method, and 2000 highly variable genes were then calculated using function *FindVariableFeatures* with “*vst*” method. Next, 3000 features which were repeatedly variable across dataset were selected using function *SelectIntegrationFeatures*, and these features were then used to find anchors using function *FindIntegrationAnchors*, and thereby multiple datasets were integrated using function *IntegrateData*.

The gene expression level was quantified with counts per million (CPM) as the UMI number of each gene divided by the total UMI number of the corresponding cell and then multiplied by 100 000. The gene expression level was transformed into log_2_(CPM + 1).

### Identification of Main Cell Types in Tissues and DEGs among Distinct Cell Types

Based on the integrated data, principal component analysis (PCA) was then performed using function *RunPCA*. To exclude the principal components (PCs) which explained very little of variance and improve the signal‐to‐noise ratio, 100 PCs (PC1–PC100) were used to perform UMAP analysis using function *RunUMAP*. Unsupervised clustering analysis was performed using function *FindNeighbors* and *FindClusters*, and clustering parameter *resolution* was set as 1 to separate cells into several clusters.

DEGs among distinct cell types were also named cell‐type specific marker genes. A Wilcoxon Rank Sum test was used to identified DEGs using function *FindAllMarkers*. Genes were considered as DEGs if log_2_‐transformed average fold change greater than 0.25, the percentage of expressed cells greater than 25% and FDR less than 0.05.

### Identification of CAF Subtypes and DEGs among Distinct Subtypes

CAFs collected from tumors and fibroblasts from normal tissues were furtherly clustered into subtypes. Gene expression data were obtained from each dataset based on identified cell names of CAFs, and distinct datasets were integrated with *Seurat* as described above and algorithm parameters remained the same. PCs 1–75 were used to perform UMAP analysis, and clustering parameter *resolution* was set as 0.8 to separate CAF subtypes. DEGs among distinct CAF subtypes were identified as same as the method described as above. GO analysis was performed using website tool *Metascape* with default parameters.^[^ ^66^ ^]^

For the identification of apCAF subtypes, integrated data generated from all fibroblasts were directly applied for clustering. DEGs among apCAF subtypes were identified as described as above.

### Survival Analysis for Distinct CAF Subtypes

Based on cell‐type specific marker genes, the survival analysis was performed in website tool *GEPIA2* to plot a Kaplan–Meier curve.^[^ ^67^ ^]^ The unrecognized genes were excluded from cell‐type specific marker genes. Genes were considered as cell‐type specific marker genes if log_2_‐transformed average fold change greater than 0.5, the percentage of expressed cells greater than 25% and FDR less than 0.05. This analysis was performed using the overall survival method, and the median expression value was set as the threshold to separate high‐expression and low‐expression cohorts.

### GSEA for CDCP1^+^FTL^+^ CAFs

GSEA was performed for KEGG pathway enrichment of CDCP1^+^FTL^+^ CAFs using JAVA‐based desktop *GSEA* software (version: 4.2.0) with CPM data.^[^ ^68^, ^69^ ^]^ The statistical method used to score hits (gene set numbers) and misses (non‐members) was set as *weighted*, and the metric for ranking genes was set as *Signal2Noise*. For reproducibility of GSEA results, the seed for randomization in the permutations process was set as *20 010 117*. All other parameters to perform GSEA were default.

### Collection of Ferroptosis‐Related Genes

The ferroptosis‐related gene sets were collected from the previous study.^[^ ^70^ ^]^

### Pseudotime Analysis to Infer the Differentiation Trajectory

Pseudotime analysis was performed to infer differentiation trajectory of CAF subtypes. Based on UMI count data, pseudotime analysis was performed using R package *Monocle* (version: 2.20.0). Function *dispersionTable* was applied to calculate the average expression and dispersion for each gene. High variable genes were then chosen if average expression greater than 0.01 and dispersion empirical greater than the dispersion fit value (set the parameter as ‘mean_expression ≥ 0.01 & dispersion_empirical ≥ 1 * dispersion_fit’), and 2503 genes were selected for the downstream analysis. A projection of single cells into a low dimensional space and pseudotime was calculated using function *reduceDimension* and *orderCells*, respectively.

When dynamic genes whose expression levels changed along pseudotime were selected, all genes were filtered and only genes with minimum expression greater than 1 and number of expressed cells greater than 10 were retained for the downstream analysis. The differential expression test was then performed using function *differentialGeneTest* with the full model parameter as *∼sm.ns(Pseudotime)*. Finally, dynamic genes were determined if the differential expression test statistical significance *p* values and adjusted *p* values both less than 0.001. The heatmap showing expression levels of dynamic genes was plotted using function *plot_pseudotime_heatmap*.

### PAGA Analysis to Infer the Differentiation Trajectory

Based on UMI count data, PAGA analysis was performed using Python package *Scanpy* (version: 1.8.1). The object generated from *Seurat* was firstly transformed into *Scanpy*‐readable *h5ad* format with function *SaveH5Seurat* and *Convert* in *Seurat*. Data normalization and log‐transformation were performed, and 3000 highly variable genes were selected for PCA. Then, based on PCA result, data from different studies were integrated with *Harmony* algorithm using function *external.pp.harmony_integrate* in *Scanpy*.^[^ ^71^ ^]^ To generate the global topology according to *Harmony* normalized data, a neighborhood graph was computed using function *pp.neighbors* with parameters *n_neighbours = 10* and *n_pcs = 30*, and force‐directed graph was drawn using function *tl.draw_graph*. Finally, the abstracted graph for quantifying the connectivity of partitions of the single‐cell graph was obtained using function *tl.paga*.

### Establishing Transcriptional Regulation Networks

To reveal the potential core transcriptional regulators in iCAFs and myCAFs 1, transcriptional regulation networks were established. Based on the human regulator database, single‐cell regulator network inference and clustering (SCENIC) algorithm was approached for network analysis using R package *SCENIC* (version: 1.2.4). For a given CAF subtype (iCAF or myCAF 1), only cell‐type specific marker genes were analyzed as input genes. After calculating the correlation of genes using UMI count data, the network was inferred with log_2_‐transformed UMI data using function *runGenie3*.^[^ ^72^ ^]^

Only regulator‐target connects with high weights greater than 0.1 were retained for visualization using software *Cytoscape*. Moreover, transcriptional regulators within dynamic genes which were obtained from pseudotime analysis were highlighted. In the visualized network plot, the node size indicated the number of connections and the line thickness indicated the weight of connections.

### Cell–Cell Interaction Analysis

Cell–cell interaction analysis of tumor and normal tissues was individually performed. Gene names were transferred into gene IDs using R package *EnsDb.Hsapiens.v79*. Based on the raw CPM data instead of log_2_‐transformed CPM data, cell–cell interactions were calculated using software *CellPhoneDB* (version: 2.0.0).^[^ ^73^, ^74^ ^]^ Only the ligand and receptor gene pair satisfying the following three criteria was considered in the downstream analysis: (1) at least 10% cells expressed that gene pair (set the parameter as “*–threshold 0.1”*), (2) the average expression level was greater than five, and (3) the enrichment of that gene pair was statistically significant (set the parameter as “*—p‐value 0.05*”). For cell–cell interactions in normal tissues, only interactions linked NFs were considered when special cell–cell interactions were identified. A ligand–receptor gene pair detected only in interactions within tumors was considered as a tumor tissue specific gene pair corresponding to newborn interactions; a ligand‐receptor gene pair detected only in interactions within normal tissues was considered as a normal tissue specific gene pair corresponding to disappeared interactions; the rest were considered as common gene pairs. GO analysis of newborn or disappeared interactions was performed using website tool *Metascape* with default parameters.

### Multiplex Immunofluorescence

Multiplex staining of was performed using TSA 7‐color kit (Akoya), according to manufacturer's instruction. The staining protocol, consisting of sequential rounds of antigen retrieval, antigen detection, and fluorescent labeling using tyramide signal amplification, was using 4‐µm‐thick sections of formalin‐fixed paraffin‐embedded (FFPE) PDAC tissues. All antibodies were first optimized via chromogenic IHC in PDAC tissue samples to ensure contextual specificity. Primary antibodies: CXCL12 (Cat# 17402‐1‐AP, Proteintech), PDPN (Cat# ab236529, Abcam), CK18 (Cat# 10830‐1‐AP, Proteintech), ISG15 (Cat# 15981‐1‐AP, Proteintech), α‐SMA (Cat# ab5694, Abcam), COLL1A1 (Cat# 72026, CST), HLA‐DR (Cat# ab20181, Abcam), CD74 (Cat# ab108393, Abcam), FTL (Cat# 10727‐1‐AP, Proteintech), CDCP1 (Cat# ab252947, Abcam), HIF‐1α (Cat# 20960‐1‐AP, Proteintech), LUM (Cat# ab168343, Abcam), CD3 (Cat# ab5690, Abcam), CD19 (Cat# ab31947, Abcam), CD16 (Cat# 16559‐1‐AP, Proteintech), TPSAB1 (Cat# ab151757, Abcam). Primary antibodies were sequentially applied, followed by HRP‐conjugated secondary antibody incubation, and tyramide signal amplification. The slides were microwave heat‐treated after each TSA operation. Nuclei were stained with DAPI after all the antigens above were labeled. The stained slides were scanned to obtain multispectral images using the Vectra Polairs (PerkinElmer).

### Isolation and Culture of Primary CAFs

Primary CAFs and NFs were isolated from three PDAC samples and paired normal tissues, which were obtained during surgery. Briefly, sheared tissues were digested with 0.5 mg/mL hyaluronidase, 1 mg/mL collagenase type IV at 37 °C for 1 h in DMEM containing 10% fetal bovine serum (FBS) and penicillin‐streptomycin. The stromal fraction was collected by centrifugation at 250 × *g* for 5 min. The remaining small tissues were incubated in DMEM medium with 20% FBS at 37 °C. Every 2–3 days, the medium was replaced, and the epithelial cells were removed via trypsinization. The remaining cells were fibroblasts. For the identification of NFs and CAFs, the expression of α‐SMA and FAP was detected among the identified markers of CAF by immunofluorescence. Further validated also performed by FACS, RT‐qPCR, and Western blot. The first through sixth passages of primary fibroblasts were used in the experiments. The isolation of CDCP1^+/−^ CAFs was performed by immunofluorescence‐activated cell sorting with surface staining for anti‐CDCP1‐PE (Cat# 324 047, Biolegend) on FACS AriaII (BD biosciences), and then collected for lysate or using for experiments immediately.

### Glycolytic Rate Assay

The cellular glycolysis were detected by monitoring ECAR with the Seahorse Bioscience Extracellular Flux Analyzer (Seahorse Bioscience Inc.). CDCP1^+/−^ CAFs were plated at a density of 8 × 10^3^ per well in an XF96 plate. Before measurements, cells were washed and then incubated with unbuffered media in a CO_2_ free incubator for 45 min. The detection of ECAR was performed in XF Base Media and the following inhibitors were added: Rotenone/Antimycin A (0.5 µM /1 µM) and 2‐deoxy‐glucose (20 µM). Measuring ECAR before and after addition of 2‐deoxy‐glucose (2‐DG), confirming the ECAR produced in the experiment was due to glycolysis, then the glycolytic ECAR was obtained. ECAR were normalized according to cell number (1 × 10^4^).

### Immunofluorescence

Cells were digested and plated on coverslips in 24‐well plates. Coverslips with cells were fixed in 4% formaldehyde in PBS for 15 min. After permeabilization with Triton X‐100 (0.2%) in PBS for 15 min, cells were blocked with PBS containing BSA (5%) for 30 min before incubation with FAP primary antibodies (Cat# AF5344, Affinity) overnight. After three separate washes, cells were incubated with a secondary antibody (Cat# ab150080, Abcam) for another 2 h and then stained with DAPI for 5 min. Coverslips were washed extensively and fixed on slides. Images were captured using a Leica laser scanning confocal microscope (Leica TCS SP8 X).

### Reverse Transcription‐PCR

RNA was extracted from indicated cells, and reverse transcription of the first‐strand cDNA was performed using a reverse‐transcription kit (Vazyme, China). The RT‐qPCR assay was conducted on the Quantstudio 6 flex system with a 2X SYBR Green mix (Yisheng, China). The data were normalized to the expression of GAPDH. The sequences of the primers were listed in Table S3 (Supporting Information).

### Flow Cytometry

For cell surface marker analysis, cells were resuspended in PBS containing 1% FBS and stained with fluorescent‐conjugated anti‐bodies against CD31 (Cat# 303 105, Biolegend), CD45 (Cat# 384 403, Biolegend), EPCAM (Cat# 324 205, Biolegend) for 30 min at 4 °C. For Vimentin (Cat# 677 809, Biolegend), Intracellular Fixation & Permeabilization Buffer Set (Invitrogen) was used according to the manufacturer's instructions. All analyses were conducted by BD Accuri C6.

### Western Blot

Protein was extracted from the cells using lysis buffer, resolved by SDS–polyacrylamide gels, and then transferred to PVDF membranes. According to the manufacturer's recommended dilution, primary antibodies against were used (1:1000). HRP conjugated secondary antibody (Proteintech) was used, and the antigen–antibody reaction was visualized by enhanced chemiluminescence assay (ECL, Tanon).

### Short Interfering RNAs Transfer

The short interfering RNA were designed and synthesized by Generalbio (Shanghai, China). Lipo3000 (Invitrogen) was used to transfect according to the manufacturer's instructions, and knock‐down efficiency was validated by qPCR. Sequence (5′‐3′) for siNC, si*FOSB*, si*NRF4A1*, si*JUN*, and si*FOS* was addressed in Table S3 (Supporting Information). The efficacy of siRNA was confirmed by a reduction of over 50% in the mRNA level of the target gene.

### Cell Contraction Assays

Collagen gel contraction assays were performed using Cell Contraction Assays Kit (Cell Biolabs). CAFs were suspended in DMEM containing 10% FBS at 2 × 10^6^ cells per mL and mixed with culture medium containing collagen I on ice at a ratio of 1: 9. The cell–collagen mixture was transferred into 24‐well plate and incubated at 37 °C for 1 h to polymerize the gel before addition of a further 1 mL DMEM containing 10% FBS. After incubation for 16 h, the culture medium was replaced with fresh medium and collagen gels were dislodged from the edge of the dish using a sterile spatula. The collagen gel size change was measured with a ruler 24 h later.

### Neutrophils Chemotaxis Assay

Neutrophils were isolated from peripheral blood of PDAC patients using Neutrophil Isolation Kit (Solarbio) according to manufacturer's protocol. Neutrophils were then seeded in the upper chamber with 5 µm pore size (Corning) in serum free DMEM and allowed to migrate to the bottom chamber of the 24‐well plate for 1 h. For the experiment, the bottom chamber was seeded with 5 × 10^4^ primary CAFs or NFs or loaded with culture media generated from human primary CAFs or NFs. Migrated neutrophils were recovered from the lower chamber and detected by measuring their ATP levels through a luminescent signal (CellTiter‐Lumi, beyotime).

### Statistical Analysis

For bioinformatics data, two‐tailed Student's *t*‐test and R software were used for statistical analysis. Quantification data were represented as means ± SD from three independent experiments. A value of *p* < 0.05 was considered to indicate statistical significance. For cell‐based experiments, biological triplicates were performed unless otherwise stated. Statistical analysis was performed using GraphPad Prism 9.0 software. Two‐tailed Student's *t*‐test were used to compare between two group (treatments versus control). One‐way ANOVA models were used to compare continuous outcomes across multiple experimental groups. Survival functions were estimated by Kaplan–Meier methods. The statistical details including the statistical tests used, exact value of *n*, and precision measures (mean ± SD or SEM) are all specified in the figure legends unless otherwise indicated.

## Conflict of Interest Statement

The authors declare no conflict of interest.

## Author Contributions

M.T., W.L., J.C., and R.L. contributed equally to this work. H.W., Y.Z., S.T., and Z.C. conceived the project and supervised the study. Y.Z. and W.L. conducted bioinformatics analysis. M.T., J.C., and R.L. designed and conducted in vitro experiments. B.Y., C.W., M.H., J.Z., Q.C., Z.Z., Z.C., and H.S. interpreted data. M.T., Y.Z., and H.W. wrote and reviewed the manuscript. All authors approved the final version of the manuscript.

## Supporting information



Supporting Information

Supplemental Table 1

Supplemental Table 2

Supplemental Table 3

## Data Availability

Research data are not shared.

## References

[1] R. L. Siegel , K. D. Miller , A. Jemal , Ca‐Cancer J. Clin. 2016, 66, 7. 26742998 10.3322/caac.21332

[2] A. N. Hosein , R. A. Brekken , A. Maitra , Nat. Rev. Gastroenterol. Hepatol. 2020, 17, 487. 32393771 10.1038/s41575-020-0300-1 PMC8284850

[3] R. E. Royal , C. Levy , K. Turner , A. Mathur , M. Hughes , U. S. Kammula , R. M. Sherry , S. L. Topalian , J. C. Yang , I. Lowy , S. A. Rosenberg , J. Immunother. 2010, 33, 828. 20842054 10.1097/CJI.0b013e3181eec14c PMC7322622

[4] J. R. Brahmer , N. Engl. J. Med. 2012, 366, 2455. 22658128 10.1056/NEJMoa1200694 PMC3563263

[5] M. H. Sherman , G. L. Beatty , Annu. Rev. Pathol.: Mech. Dis. 2023, 18, 123. 10.1146/annurev-pathmechdis-031621-024600 PMC9877114 36130070

[6] P. P. Provenzano , C. Cuevas , A. E. Chang , V. K. Goel , D. D. Von Hoff , S. R. Hingorani , Cancer Cell 2012, 21, 418. 22439937 10.1016/j.ccr.2012.01.007 PMC3371414

[7] E. Sahai , I. Astsaturov , E. Cukierman , D. G. DeNardo , M. Egeblad , R. M. Evans , D. Fearon , F. R. Greten , S. R. Hingorani , T. Hunter , R. O. Hynes , R. K. Jain , T. Janowitz , C. Jorgensen , A. C. Kimmelman , M. G. Kolonin , R. G. Maki , R. S. Powers , E. Puré , D. C. Ramirez , R. Scherz‐Shouval , M. H. Sherman , S. Stewart , T. D. Tlsty , D. A. Tuveson , F. M. Watt , V. Weaver , A. T. Weeraratna , Z. Werb , Nat. Rev. Cancer 2020, 20, 174. 31980749 10.1038/s41568-019-0238-1 PMC7046529

[8] D. Hanahan , L. M. Coussens , Cancer Cell 2012, 21, 309. 22439926 10.1016/j.ccr.2012.02.022

[9] C. K. Pallangyo , P. K. Ziegler , F. R. Greten , J. Exp. Med. 2015, 212, 2253. 26621452 10.1084/jem.20150576 PMC4689166

[10] S. Su , J. Chen , H. Yao , J. Liu , S. Yu , L. Lao , M. Wang , M. Luo , Y. Xing , F. Chen , D.i Huang , J. Zhao , L. Yang , D. Liao , F. Su , M. Li , Q. Liu , E. Song , Cell 2018, 172, 841. 29395328 10.1016/j.cell.2018.01.009

[11] A. Costa , Y. Kieffer , A. Scholer‐Dahirel , F. Pelon , B. Bourachot , M. Cardon , P. Sirven , I. Magagna , L. Fuhrmann , C. Bernard , C. Bonneau , M. Kondratova , I. Kuperstein , A. Zinovyev , A.‐M. Givel , M.‐C. Parrini , V. Soumelis , A. Vincent‐Salomon , F. Mechta‐Grigoriou , Cancer Cell 2018, 33, 463. 29455927 10.1016/j.ccell.2018.01.011

[12] C. Feig , J. O. Jones , M. Kraman , R. J. B. Wells , A. Deonarine , D. S. Chan , C. M. Connell , E. W. Roberts , Q.i Zhao , O. L. Caballero , S. A. Teichmann , T. Janowitz , D. I. Jodrell , D. A. Tuveson , D. T. Fearon , Proc. Natl. Acad. Sci. U. S. A. 2013, 110, 20212. 24277834 10.1073/pnas.1320318110 PMC3864274

[13] H. Jiang , S. Hegde , B. L. Knolhoff , Y.u Zhu , J. M. Herndon , M. A. Meyer , T. M. Nywening , W. G. Hawkins , I. M. Shapiro , D. T. Weaver , J. A. Pachter , A. Wang‐Gillam , D. G. DeNardo , Nat. Med. 2016, 22, 851. 27376576 10.1038/nm.4123 PMC4935930

[14] D. V. T. Catenacci , M. R. Junttila , T. Karrison , N. Bahary , M. N. Horiba , S. R. Nattam , R. Marsh , J. Wallace , M. Kozloff , L. Rajdev , D. Cohen , J. Wade , B. Sleckman , H.‐J. Lenz , P. Stiff , P. Kumar , P. Xu , L. Henderson , N. Takebe , R. Salgia , X.i Wang , W. M. Stadler , F. J. de Sauvage , H. L. Kindler , J. Clin. Oncol. 2015, 33, 4284. 26527777 10.1200/JCO.2015.62.8719 PMC4678179

[15] E. J. Kim , V. Sahai , E. V. Abel , K. A. Griffith , J. K. Greenson , N. Takebe , G. N. Khan , J. L. Blau , R. Craig , U. G. Balis , M. M. Zalupski , D. M. Simeone , Clin. Cancer Res. 2014, 20, 5937. 25278454 10.1158/1078-0432.CCR-14-1269 PMC4254161

[16] B. C. Özdemir , T. Pentcheva‐Hoang , J. L. Carstens , X. Zheng , C.‐C. Wu , T. R. Simpson , H. Laklai , H. Sugimoto , C. Kahlert , S. V. Novitskiy , A. De Jesus‐Acosta , P. Sharma , P. Heidari , U. Mahmood , L. Chin , H. L. Moses , V. M. Weaver , A. Maitra , J. P. Allison , V. S. LeBleu , R. Kalluri , Cancer Cell 2014, 25, 719. 24856586 10.1016/j.ccr.2014.04.005 PMC4180632

[17] R. Kalluri , Nat. Rev. Cancer 2016, 16, 582. 27550820 10.1038/nrc.2016.73

[18] E. Elyada , M. Bolisetty , P. Laise , W. F. Flynn , E. T. Courtois , R. A. Burkhart , J. A. Teinor , P. Belleau , G. Biffi , M. S. Lucito , S. Sivajothi , T. D. Armstrong , D. D. Engle , K. H. Yu , Y. Hao , C. L. Wolfgang , Y. Park , J. Preall , E. M. Jaffee , A. Califano , P. Robson , D. A. Tuveson , Cancer Discovery 2019, 9, 1102. 31197017 10.1158/2159-8290.CD-19-0094 PMC6727976

[19] G. Biffi , T. E. Oni , B. Spielman , Y. Hao , E. Elyada , Y. Park , J. Preall , D. A. Tuveson , Cancer Discovery 2019, 9, 282. 30366930 10.1158/2159-8290.CD-18-0710 PMC6368881

[20] D. Öhlund , A. Handly‐Santana , G. Biffi , E. Elyada , A. S. Almeida , M. Ponz‐Sarvise , V. Corbo , T. E. Oni , S. A. Hearn , E. J. Lee , I. I. C. Chio , C.‐I.l Hwang , H. Tiriac , L. A. Baker , D. D. Engle , C. Feig , A. Kultti , M. Egeblad , D. T. Fearon , J. M. Crawford , H. Clevers , Y. Park , D. A. Tuveson , J. Exp. Med. 2017, 214, 579. 28232471 10.1084/jem.20162024 PMC5339682

[21] J. Peng , B.‐F.a Sun , C.‐Y. Chen , J.‐Y.i Zhou , Y.u‐S. Chen , H. Chen , L. Liu , D. Huang , J. Jiang , G.‐S. Cui , Y. Yang , W. Wang , D. Guo , M. Dai , J. Guo , T. Zhang , Q. Liao , Y.i Liu , Y.‐L. Zhao , D.a‐L.i Han , Y. Zhao , Y.‐G. Yang , W. Wu , Cell Res. 2019, 29, 725. 31273297 10.1038/s41422-019-0195-y PMC6796938

[22] W. Lin , P. Noel , E. H. Borazanci , J. Lee , A. Amini , I. W. Han , J. S. Heo , G. S. Jameson , C. Fraser , M. Steinbach , Y. Woo , Y. Fong , D. Cridebring , D. D. Von Hoff , J. O.h Park , H. Han , Genome Med. 2020, 12, 80. 32988401 10.1186/s13073-020-00776-9 PMC7523332

[23] N. G. Steele , E. S. Carpenter , S. B. Kemp , V. R. Sirihorachai , S. The , L. Delrosario , J. Lazarus , E.l‐A. D. Amir , V. Gunchick , C. Espinoza , S. Bell , L. Harris , F. Lima , V. Irizarry‐Negron , D. Paglia , J. Macchia , A. K. Y. Chu , H. Schofield , E.‐J. Wamsteker , R. Kwon , A. Schulman , A. Prabhu , R. Law , A. Sondhi , J. Yu , A. Patel , K. Donahue , H. Nathan , C. Cho , M. A. Anderson , et al., Nat. Cancer 2020, 1, 1097. 34296197 10.1038/s43018-020-00121-4 PMC8294470

[24] Y. Schlesinger , O. Yosefov‐Levi , D. Kolodkin‐Gal , R. Z. Granit , L. Peters , R. Kalifa , L. Xia , A. Nasereddin , I. Shiff , O. Amran , Y. Nevo , S. Elgavish , K. Atlan , G. Zamir , O. Parnas , Nat. Commun. 2020, 11, 4516. 32908137 10.1038/s41467-020-18207-z PMC7481797

[25] H. Yao , Z. Yang , Z. Liu , X. Miao , L. Yang , D. Li , Q. Zou , Y. Yuan , Cancer Biomarkers 2016, 17, 397. 27689616 10.3233/CBM-160655 PMC13020517

[26] K. Hildner , B. T. Edelson , W. E. Purtha , M. Diamond , H. Matsushita , M. Kohyama , B. Calderon , B. U. Schraml , E. R. Unanue , M. S. Diamond , R. D. Schreiber , T. L. Murphy , K. M. Murphy , Science 2008, 322, 1097. 19008445 10.1126/science.1164206 PMC2756611

[27] Z. Chen , L. Zhou , L. Liu , Y. Hou , M. Xiong , Y.u Yang , J. Hu , K.e Chen , Nat. Commun. 2020, 11, 5077. 33033240 10.1038/s41467-020-18916-5 PMC7545162

[28] J. T. Shieh , J. A. Tintos‐Hernandez , C. N. Murali , M. Penon‐Portmann , M. Flores‐Mendez , A. Santana , J. A. Bulos , K. Du , L. Dupuis , N. Damseh , R. Mendoza‐Londoño , C. Berera , J. Lee , J. J. Phillips , C. A. P. F. Alves , I. J. Dmochowski , X. R. Ortiz‐González , HGG Adv. 2023, 4, 100236. 37660254 10.1016/j.xhgg.2023.100236 PMC10510067

[29] H. Bayır , S. J. Dixon , Y. Y. Tyurina , J. A. Kellum , V. E. Kagan , Nat. Rev. Nephrol. 2023, 19, 315. 36922653 10.1038/s41581-023-00689-x

[30] J. Liu , L. Gao , N. Zhan , P. Xu , J. Yang , F. Yuan , Y. Xu , Q. Cai , R. Geng , Q. Chen , J. Exp. Clin. Cancer Res. 2020, 39, 137. 32677981 10.1186/s13046-020-01641-8 PMC7364815

[31] W. Hu , C. Zhou , Q. Jing , Y. Li , J. Yang , C. Yang , L. Wang , J. Hu , H. Li , H. Wang , C. Yuan , Y.i Zhou , X. Ren , X. Tong , J. Du , Y. Wang , Cancer Cell Int. 2021, 21, 709. 34965856 10.1186/s12935-021-02420-x PMC8717654

[32] P. Koppula , L. Zhuang , B. Gan , Protein Cell 2020, 12, 599. 33000412 10.1007/s13238-020-00789-5 PMC8310547

[33] S. Doll , B. Proneth , Y. Y. Tyurina , E. Panzilius , S. Kobayashi , I. Ingold , M. Irmler , J. Beckers , M. Aichler , A. Walch , H. Prokisch , D. Trümbach , G. Mao , F. Qu , H. Bayir , J. Füllekrug , C. H. Scheel , W. Wurst , J. A. Schick , V. E. Kagan , J. P. F. Angeli , M. Conrad , Nat. Chem. Biol. 2017, 13, 91. 27842070 10.1038/nchembio.2239 PMC5610546

[34] Z. Yang , W. Su , X. Wei , S. Qu , D. Zhao , J. Zhou , Y. Wang , Q. Guan , C. Qin , J. Xiang , K. Zen , B. Yao , Cell Rep. 2023, 42, 112945. 37542723 10.1016/j.celrep.2023.112945

[35] B. M. Emerling , C. H. Benes , G. Poulogiannis , E. L. Bell , K. Courtney , H. Liu , R. Choo‐Wing , G. Bellinger , K. S. Tsukazawa , V. Brown , S. Signoretti , S. P. Soltoff , L. C. Cantley , Proc. Natl. Acad. Sci. U. S. A. 2013, 110, 3483. 23378636 10.1073/pnas.1222435110 PMC3587206

[36] F. A. Wolf , P. Angerer , F. J. Theis , Genome Biol. 2018, 19, 15. 29409532 10.1186/s13059-017-1382-0 PMC5802054

[37] K. Kretzschmar , C. Weber , R. R. Driskell , E. Calonje , F. M. Watt , Cell Rep. 2016, 14, 269. 26771241 10.1016/j.celrep.2015.12.041 PMC4713864

[38] A. E. Karnoub , A. B. Dash , A. P. Vo , A. Sullivan , M. W. Brooks , G. W. Bell , A. L. Richardson , K. Polyak , R. Tubo , R. A. Weinberg , Nature 2007, 449, 557. 17914389 10.1038/nature06188

[39] Y. Raz , N. Cohen , O. Shani , R. E. Bell , S. V. Novitskiy , L. Abramovitz , C. Levy , M. Milyavsky , L. Leider‐Trejo , H. L. Moses , D. Grisaru , N. Erez , J. Exp. Med. 2018, 215, 3075. 30470719 10.1084/jem.20180818 PMC6279405

[40] M. Mastrogiannaki , B. M. Lichtenberger , A. Reimer , C. A. Collins , R. R. Driskell , F. M. Watt , J. Invest. Dermatol. 2016, 136, 1130. 26902921 10.1016/j.jid.2016.01.036 PMC4874948

[41] L. Bochet , C. Lehuédé , S. Dauvillier , Y. Y. Wang , B. Dirat , V. Laurent , C. Dray , R. Guiet , I. Maridonneau‐Parini , S. Le Gonidec , B. Couderc , G. Escourrou , P. Valet , C. Muller , Cancer Res. 2013, 73, 5657. 23903958 10.1158/0008-5472.CAN-13-0530

[42] Q. Li , X. Lv , C. Han , Y.u Kong , Z. Dai , D. Huo , T. Li , D. Li , W. Li , X. Wang , Q. Zhao , J. Ming , W. Yang , Y. Chen , X. Wu , Theranostics 2022, 12, 7491. 36438487 10.7150/thno.75853 PMC9691365

[43] P. Angel , M. Karin , Biochim. Biophys. Acta, Rev. Cancer 1991, 1072, 129. 10.1016/0304-419x(91)90011-9 1751545

[44] T. Liu , F. Gonzalez De Los Santos , M. Hirsch , Z. Wu , S. H. Phan , Am. J. Respir. Cell Mol. Biol. 2021, 65, 489. 34107237 10.1165/rcmb.2020-0499OC PMC8641847

[45] F. Wu , J. Yang , J. Liu , Y.e Wang , J. Mu , Q. Zeng , S. Deng , H. Zhou , Signal Transduction Targeted Ther. 2021, 6, 218. 10.1038/s41392-021-00641-0 PMC8190181 34108441

[46] L. L. Tran , T. Dang , R. Thomas , D. R. Rowley , Stem Cells 2021, 39, 1766. 34520582 10.1002/stem.3455 PMC8963131

[47] T. Tsujikawa , S. Kumar , R. N. Borkar , V. Azimi , G. Thibault , Y. H. Chang , A. Balter , R. Kawashima , G. Choe , D. Sauer , E. El Rassi , D. R. Clayburgh , M. F. Kulesz‐Martin , E. R. Lutz , L. Zheng , E. M. Jaffee , P. Leyshock , A. A. Margolin , M. Mori , J. W. Gray , P. W. Flint , L. M. Coussens , Cell Rep. 2017, 19, 203. 28380359 10.1016/j.celrep.2017.03.037 PMC5564306

[48] E. N. Arwert , E. L. Milford , A. Rullan , S. Derzsi , S. Hooper , T. Kato , D. Mansfield , A. Melcher , K. J. Harrington , E. Sahai , Nat. Cell Biol. 2020, 22, 758. 32483388 10.1038/s41556-020-0527-7 PMC7611090

[49] U. E. Martinez‐Outschoorn , M. P. Lisanti , F. Sotgia , Semin. Cancer Biol. 2014, 25, 47. 24486645 10.1016/j.semcancer.2014.01.005

[50] Y.u Wang , Y. Liang , H. Xu , X. Zhang , T. Mao , J. Cui , J. Yao , Y. Wang , F. Jiao , X. Xiao , J. Hu , Q. Xia , X. Zhang , X. Wang , Y. Sun , D. Fu , L. Shen , X. Xu , J. Xue , L. Wang , Cell Discovery 2021, 7, 36. 34035226 10.1038/s41421-021-00271-4 PMC8149399

[51] X. Jiang , B. R. Stockwell , M. Conrad , Nat. Rev. Mol. Cell Biol. 2021, 22, 266. 33495651 10.1038/s41580-020-00324-8 PMC8142022

[52] G. Lei , L. Zhuang , B. Gan , Nat. Rev. Cancer 2022, 22, 381. 35338310 10.1038/s41568-022-00459-0 PMC10243716

[53] Y. Zhu , S. Fang , B. Fan , K. Xu , L. Xu , L. Wang , L. Zhu , C. Chen , R. Wu , J. Ni , J. Wang , Theranostics 2024, 14, 1683. 38389839 10.7150/thno.89805 PMC10879865

[54] H. Huang , Z. Wang , Y. Zhang , R. N. Pradhan , D. Ganguly , R. Chandra , G. Murimwa , S. Wright , X. Gu , R. Maddipati , S. Müller , S. J. Turley , R. A. Brekken , Cancer Cell 2022, 40, 656. 35523176 10.1016/j.ccell.2022.04.011 PMC9197998

[55] D. Kerdidani , E. Aerakis , K.‐M. Verrou , I. Angelidis , K. Douka , M.‐A. Maniou , P. Stamoulis , K. Goudevenou , A. Prados , C. Tzaferis , V. Ntafis , I. Vamvakaris , E. Kaniaris , K. Vachlas , E. Sepsas , A. Koutsopoulos , K. Potaris , M. Tsoumakidou , J. Exp. Med. 2022, 219, e20210815. 35029648 10.1084/jem.20210815 PMC8764966

[56] M. Bartoschek , N. Oskolkov , M. Bocci , J. Lövrot , C. Larsson , M. Sommarin , C. D. Madsen , D. Lindgren , G. Pekar , G. Karlsson , M. Ringnér , J. Bergh , Å. Björklund , K. Pietras , Nat. Commun. 2018, 9, 5150. 30514914 10.1038/s41467-018-07582-3 PMC6279758

[57] L. Mazzeo , S. Ghosh , E. Di Cicco , J. Isma , D. Tavernari , A. Samarkina , P. Ostano , M. K. Youssef , C. Simon , G. P. Dotto , Nat. Commun. 2024, 15, 1038. 38310103 10.1038/s41467-024-45308-w PMC10838290

[58] D. E. Sullivan , M. Ferris , H. Nguyen , E. Abboud , A. R. Brody , J. Cell. Mol. Med. 2009, 13, 1866. 20141610 10.1111/j.1582-4934.2008.00647.x PMC2855747

[59] J. E. Murphy , J. Y. Wo , D. P. Ryan , J. W. Clark , W. Jiang , B. Y. Yeap , L. C. Drapek , L. Ly , C. V. Baglini , L. S. Blaszkowsky , C. R. Ferrone , A. R. Parikh , C. D. Weekes , R. D. Nipp , E. L. Kwak , J. N. Allen , R. B. Corcoran , D. T. Ting , J. E. Faris , A. X. Zhu , L. Goyal , D. L. Berger , M. Qadan , K. D. Lillemoe , N. Talele , R. K. Jain , T. F. DeLaney , D. G. Duda , Y. Boucher , C. Fernández‐Del Castillo , et al., JAMA Oncol. 2019, 5, 1020. 31145418 10.1001/jamaoncol.2019.0892 PMC6547247

[60] T. M. Nywening , B. A. Belt , D. R. Cullinan , R. Z. Panni , B. J. Han , D. E. Sanford , R. C. Jacobs , J. Ye , A. A. Patel , W. E. Gillanders , R. C. Fields , D. G. DeNardo , W. G. Hawkins , P. Goedegebuure , D. C. Linehan , Gut 2018, 67, 1112. 29196437 10.1136/gutjnl-2017-313738 PMC5969359

[61] Y.u Zhu , B. L. Knolhoff , M. A. Meyer , T. M. Nywening , B. L. West , J. Luo , A. Wang‐Gillam , S. P. Goedegebuure , D. C. Linehan , D. G. DeNardo , Cancer Res. 2014, 74, 5057. 25082815 10.1158/0008-5472.CAN-13-3723 PMC4182950

[62] A. J. Gentles , A. M. Newman , C. L. Liu , S. V. Bratman , W. Feng , D. Kim , V. S. Nair , Y. Xu , A. Khuong , C. D. Hoang , M. Diehn , R. B. West , S. K. Plevritis , A. A. Alizadeh , Nat. Med. 2015, 21, 938. 26193342 10.1038/nm.3909 PMC4852857

[63] C. W. Steele , S. A. Karim , J. D. G. Leach , P. Bailey , R. Upstill‐Goddard , L. Rishi , M. Foth , S. Bryson , K. McDaid , Z. Wilson , C. Eberlein , J. B. Candido , M. Clarke , C. Nixon , J. Connelly , N. Jamieson , C. R. Carter , F. Balkwill , D. K. Chang , T. R. J. Evans , D. Strathdee , A. V. Biankin , R. J. B. Nibbs , S. T. Barry , O. J. Sansom , J. P. Morton , Cancer Cell 2016, 29, 832. 27265504 10.1016/j.ccell.2016.04.014 PMC4912354

[64] S. R. Nielsen , J. E. Strøbech , E. R. Horton , R. Jackstadt , A. Laitala , M. C. Bravo , G. Maltese , A. R. D. Jensen , R. Reuten , M. Rafaeva , S. A. Karim , C.‐I.l Hwang , L. Arnes , D. A. Tuveson , O. J. Sansom , J. P. Morton , J. T. Erler , Nat. Commun. 2021, 12, 3414. 34099731 10.1038/s41467-021-23731-7 PMC8184753

[65] R. Satija , J. A. Farrell , D. Gennert , A. F. Schier , A. Regev , Nat. Biotechnol. 2015, 33, 495. 25867923 10.1038/nbt.3192 PMC4430369

[66] Y. Zhou , B. Zhou , L. Pache , M. Chang , A. H. Khodabakhshi , O. Tanaseichuk , C. Benner , S. K. Chanda , Nat. Commun. 2019, 10, 1523. 30944313 10.1038/s41467-019-09234-6 PMC6447622

[67] Z. Tang , B. Kang , C. Li , T. Chen , Z. Zhang , Nucleic Acids Res. 2019, 47, W556. 31114875 10.1093/nar/gkz430 PMC6602440

[68] V. K. Mootha , C. M. Lindgren , K.‐F. Eriksson , A. Subramanian , S. Sihag , J. Lehar , P. Puigserver , E. Carlsson , M. Ridderstråle , E. Laurila , N. Houstis , M. J. Daly , N. Patterson , J. P. Mesirov , T. R. Golub , P. Tamayo , B. Spiegelman , E. S. Lander , J. N. Hirschhorn , D. Altshuler , L. C. Groop , Nat. Genet. 2003, 34, 267. 12808457 10.1038/ng1180

[69] A. Subramanian , P. Tamayo , V. K. Mootha , S. Mukherjee , B. L. Ebert , M. A. Gillette , A. Paulovich , S. L. Pomeroy , T. R. Golub , E. S. Lander , J. P. Mesirov , Proc. Natl. Acad. Sci. U. S. A. 2005, 102, 15545. 16199517 10.1073/pnas.0506580102 PMC1239896

[70] F. Yang , Y. Xiao , J.‐H. Ding , X.i Jin , D. Ma , D.‐Q. Li , J.‐X. Shi , W. Huang , Y.‐P. Wang , Y.‐Z. Jiang , Z.‐M. Shao , Cell Metab. 2023, 35, 84. 36257316 10.1016/j.cmet.2022.09.021

[71] I. Korsunsky , N. Millard , J. Fan , K. Slowikowski , F. Zhang , K. Wei , Y. Baglaenko , M. Brenner , P.‐R. Loh , S. Raychaudhuri , Nat. Methods 2019, 16, 1289. 31740819 10.1038/s41592-019-0619-0 PMC6884693

[72] S. Chen , J. C. Mar , BMC Bioinf. 2018, 19, 232. 10.1186/s12859-018-2217-z PMC6006753 29914350

[73] M. Efremova , M. Vento‐Tormo , S. A. Teichmann , R. Vento‐Tormo , Nat. Protoc. 2020, 15, 1484. 32103204 10.1038/s41596-020-0292-x

[74] R. Vento‐Tormo , M. Efremova , R. A. Botting , M. Y. Turco , M. Vento‐Tormo , K. B. Meyer , J.‐E. Park , E. Stephenson , K. Polanski , A. Goncalves , L. Gardner , S. Holmqvist , J. Henriksson , A. Zou , A. M. Sharkey , B. Millar , B. Innes , L. Wood , A. Wilbrey‐Clark , R. P. Payne , M. A. Ivarsson , S. Lisgo , A. Filby , D. H. Rowitch , J. N. Bulmer , G. J. Wright , M. J. T. Stubbington , M. Haniffa , A. Moffett , S. A. Teichmann , Nature 2018, 563, 347. 30429548 10.1038/s41586-018-0698-6 PMC7612850

