# Supplementary material for: Transcriptome Landscape of Cancer‐Associated Fibroblasts in Human PDAC
Source: Adv Sci (Weinh). 2025 Feb 28;12(20):2415196. doi: 10.1002/advs.202415196 (PMC12120754; doi:10.1002/advs.202415196)
Supplement: Supplementary file 1 — Supporting Information [file ADVS-12-2415196-s002.pdf]

ADVANCED SCIENCE
Open Access

Supporting Information

for *Adv. Sci.*, DOI 10.1002/advs.202415196

Transcriptome Landscape of Cancer-Associated Fibroblasts in Human PDAC

Mengyu Tao, Wenting Liu, Jianhua Chen, Rujiao Liu, Jianling Zou, Bo Yu, Chenchen Wang, Mingzhu Huang, Qingjian Chen, Zhe Zhang, Zhiyu Chen, Haoyu Sun, Cheng Zhou, Shuguang Tan*, Yuxuan Zheng* and Hongxia Wang**

Supplementary Information

Transcriptome landscape of cancer associated fibroblasts in human PDAC

Mengyu Tao, Wenting Liu, Jianhua Chen, Rujiao Liu, Jianling Zou, Bo Yu, Chenchen Wang, Mingzhu Huang, Qingjian Chen, Zhe Zhang, Zhiyu Chen, Haoyu Sun, Cheng Zhou[#], Shuguang Tan[#], Yuxuan Zheng7[#], Hongxia Wang[#]

Supplementary Figures 1-8

Figure S1. Quality control information of collected single-cell RNA-seq data.

Figure S2. Identification CAF subtypes.

Figure S3. Identification and functional role of CDCP1^{+}FTL^{+} CAF in PDAC.

Figure S4. Identification of apCAF subtypes and immune cells co-expressed CAF marker genes.

Figure S5. Pesudotime analysis of distinct CAF subtypes.

Figure S6. AP-1 members triggering the malignant phenotype conversion of NFs.

Figure S7. IFNγ contributing to the fate transformation from classic myCAFs to iCAFs

Figure S8. Cell-cell interactions between CAF subtypes and other cells.

Supplementary Tables 1-3

Table S1. Cell information.

Table S2. DEGs and monocle gene groups.

Table S3. Primer and siRNA sequence.

Figure S1

A

Data label	Platform	Data accession	Sample	Cell number	Reference
Steele et al., 2020	10×	GSE155698	PDAC tissue (n = 16)<br>Normal tissue (n = 3)	48,279	Multimodal mapping of the tumor and peripheral blood immune landscape in human pancreatic cancer (Nature Cancer, 2020)
Schlesinger et al., 2020	10×	GSE141017	PDAC tissue (mixed)	5,018	Single-cell transcriptomes of pancreatic preinvasive lesions and cancer reveal acinar metaplastic cells' heterogeneity (Nature Communications, 2020)
Lin et al., 2020	10×	GSE154778	PDAC tissue (n = 10)<br>Metastatic tissue (n = 6)	15,076	Single-cell transcriptome analysis of tumor and stromal compartments of pancreatic ductal adenocarcinoma primary tumors and metastatic lesions (Genome Medicine, 2020)
Elyada et al., 2019	10×	Request for authors	PDAC tissue (n = 6)<br>Normal tissue (n = 2)	20,369	Cross-species single-cell analysis of pancreatic ductal adenocarcinoma reveals antigen-presenting cancer-associated fibroblasts (Cancer Discovery, 2019)
Peng et al., 2019	10×	Request for authors	PDAC tissue (n = 24)<br>Normal tissue (n = 11)	57,443	Single-cell RNA-seq highlights intra-tumoral heterogeneity and malignant progression in pancreatic ductal adenocarcinoma (Cell Research, 2019)

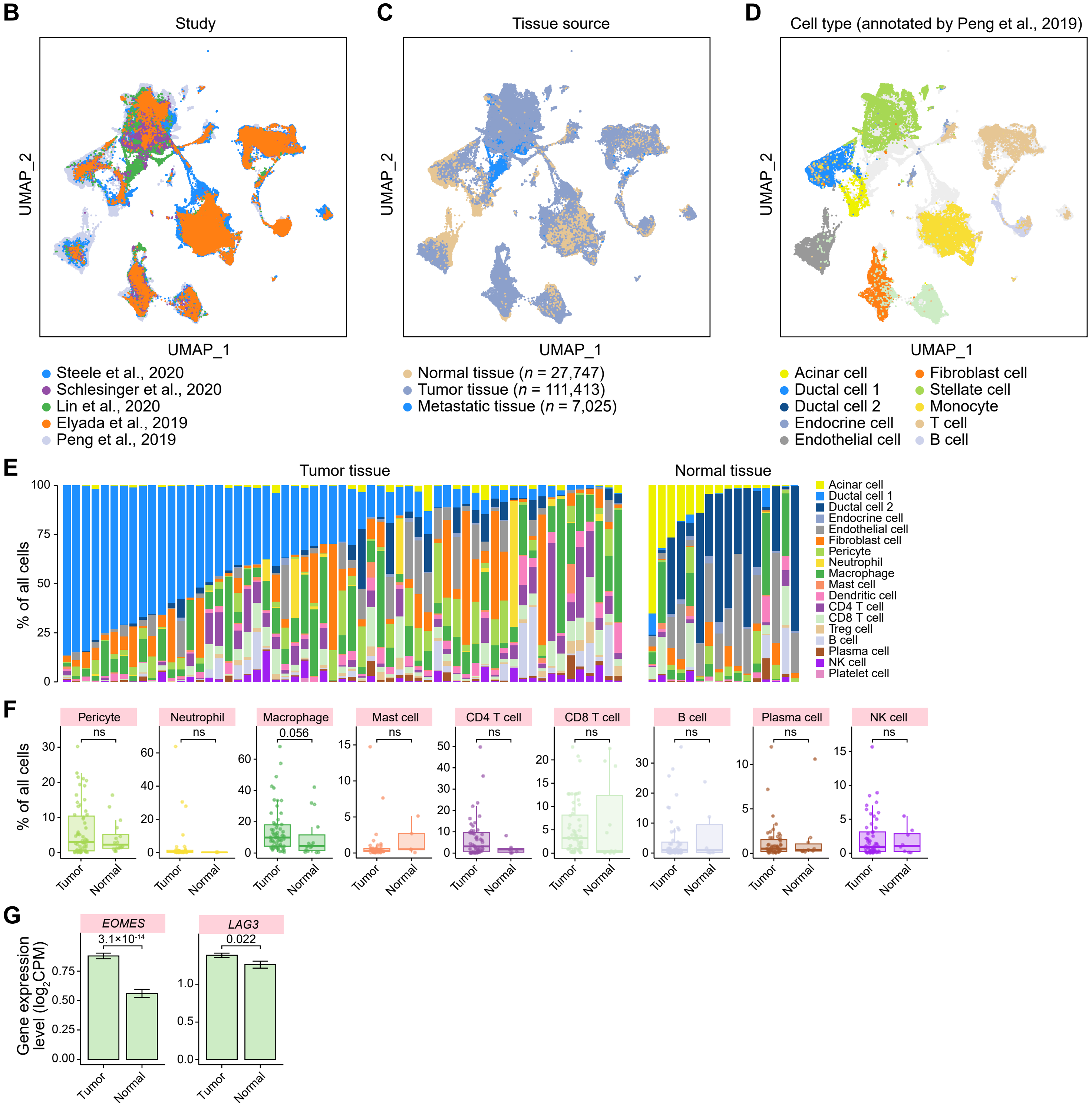

Figure S1. Quality control information of collected single-cell RNA-seq data.

(A) Table showing the detail information of collected single-cell RNA-seq data.

(B and C) UMAP plots showing the clustering of all cells. The dot color indicates data sources (B) or tissue sources (C). The number of cells in each data source or tissue source is indicated in brackets, respectively.

(D) UMAP plot showing the clustering of all cells. The dot color indicates cell types which are directly obtained from the previous study (Peng et al., 2019).

(E) Stacked bar plots showing the composition of distinct cell types in tumor (left) and normal (right) tissues.

(F) Boxplots showing the percentage of representative cell types among all cells from tumor and normal tissues. The two-tailed Wilcoxon-ranked *P* value is indicated.

(G) Bar plots showing the expression level of exhausted markers in CD8 T cells. The two-tailed Student's *t*-test *P* value is indicated. Data are shown with the mean value ± SEM.

Figure S2

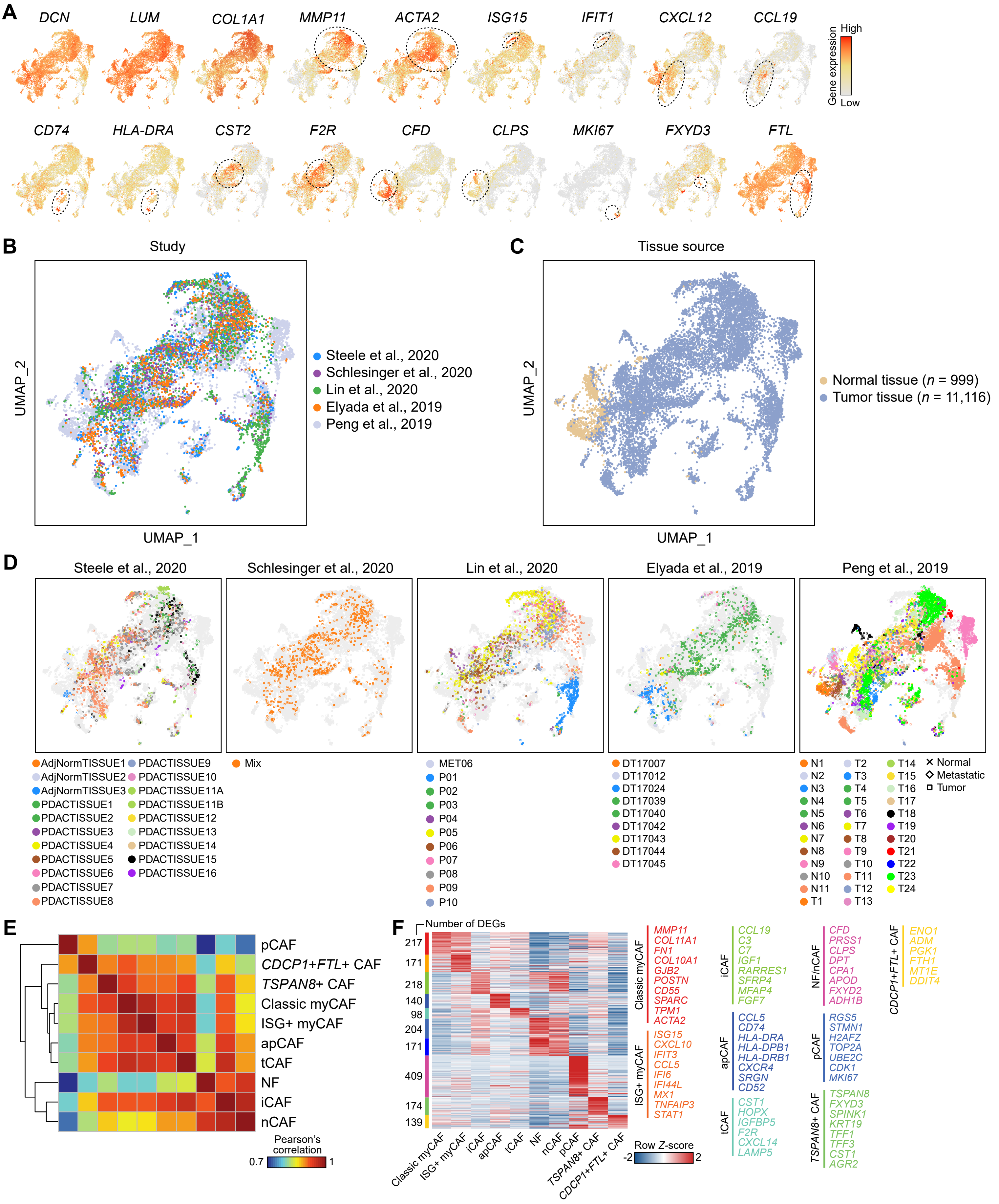
A
DCN
LUM
COL1A1
MMP11
ACTA2
ISG15
IFIT1
CXCL12
CCL19
High
Gene expression
Low
CD74
HLA-DRA
CST2
F2R
CFD
CLPS
MKI67
FXYD3
FTL
B
Study
UMAP_2
UMAP_1
Steele et al., 2020
Schlesinger et al., 2020
Lin et al., 2020
Elyada et al., 2019
Peng et al., 2019
C
Tissue source
UMAP_2
UMAP_1
Normal tissue (n = 999)
Tumor tissue (n = 11,116)
D
Steele et al., 2020
Schlesinger et al., 2020
Lin et al., 2020
Elyada et al., 2019
Peng et al., 2019
AdjNormTISSUE1
AdjNormTISSUE2
AdjNormTISSUE3
PDACTISSUE1
PDACTISSUE2
PDACTISSUE3
PDACTISSUE4
PDACTISSUE5
PDACTISSUE6
PDACTISSUE7
PDACTISSUE8
PDACTISSUE9
PDACTISSUE10
PDACTISSUE11A
PDACTISSUE11B
PDACTISSUE12
PDACTISSUE13
PDACTISSUE14
PDACTISSUE15
PDACTISSUE16
Mix
MET06
P01
P02
P03
P04
P05
P06
P07
P08
P09
P10
DT17007
DT17012
DT17024
DT17039
DT17040
DT17042
DT17043
DT17044
DT17045
N1
N2
N3
N4
N5
N6
N7
N8
N9
N10
N11
T1
T2
T3
T4
T5
T6
T7
T8
T9
T10
T11
T12
T13
T14
T15
T16
T17
T18
T19
T20
T21
T22
T23
T24
Normal
Metastatic
Tumor
E
pCAF
CDCP1+FTL+ CAF
TSPAN8+ CAF
Classic myCAF
ISG+ myCAF
apCAF
tCAF
NF
iCAF
nCAF
Pearson's correlation
0.7
1
F
Number of DEGs
217
171
218
140
98
204
171
409
174
139
Classic myCAF
ISG+ myCAF
iCAF
apCAF
tCAF
NF
nCAF
pCAF
TSPAN8+ CAF
CDCP1+FTL+ CAF
Row Z-score
-2
2
Classic myCAF
MMP11
COL11A1
FN1
COL10A1
GJB2
POSTN
CD55
SPARC
TPM1
ACTA2
ISG+ myCAF
ISG15
CXCL10
IFIT3
CCL5
IFI6
IFI44L
MX1
TNFAIP3
STAT1
iCAF
CCL19
C3
C7
IGF1
RARRES1
SFRP4
MFAP4
FGF7
apCAF
CCL5
CD74
HLA-DRA
HLA-DPB1
HLA-DRB1
CXCR4
SRGN
CD52
tCAF
CST1
HOPX
IGFBP5
F2R
CXCL14
LAMP5
NF/nCAF
CFD
PRSS1
CLPS
DPT
CPA1
APOD
FXYD2
ADH1B
pCAF
RGS5
STMN1
H2AFZ
TOP2A
UBE2C
CDK1
MKI67
TSPAN8+ CAF
TSPAN8
FXYD3
SPINK1
KRT19
TFF1
TFF3
CST1
AGR2
CDCP1+FTL+ CAF
ENO1
ADM
PGK1
FTH1
MT1E
DDIT4

Figure S2. Identification CAF subtypes.

(A) UMAP plots showing the expression level of pan-CAF marker genes and CAF subtype signature genes.

(B and C) UMAP plots showing the clustering of fibroblasts collected from normal or tumor tissues. The dot color indicates data sources (B) or tissue sources (C). The number of cells in each tissue source is indicated in brackets.

(D) UMAP plot showing the clustering of fibroblasts collected from normal or tumor tissues. The dot color indicates samples in each study.

(E) Heatmap showing the Pearson's correlation coefficient of distinct CAF subtypes in pairwise manner.

(F) Heatmap showing the expression level of DEGs in each CAF subtype. Values are scaled in each gene with *Z*-score. The number of DEGs in each subtype is indicated in the left, and represented DEGs are indicated in the right.

Figure S3

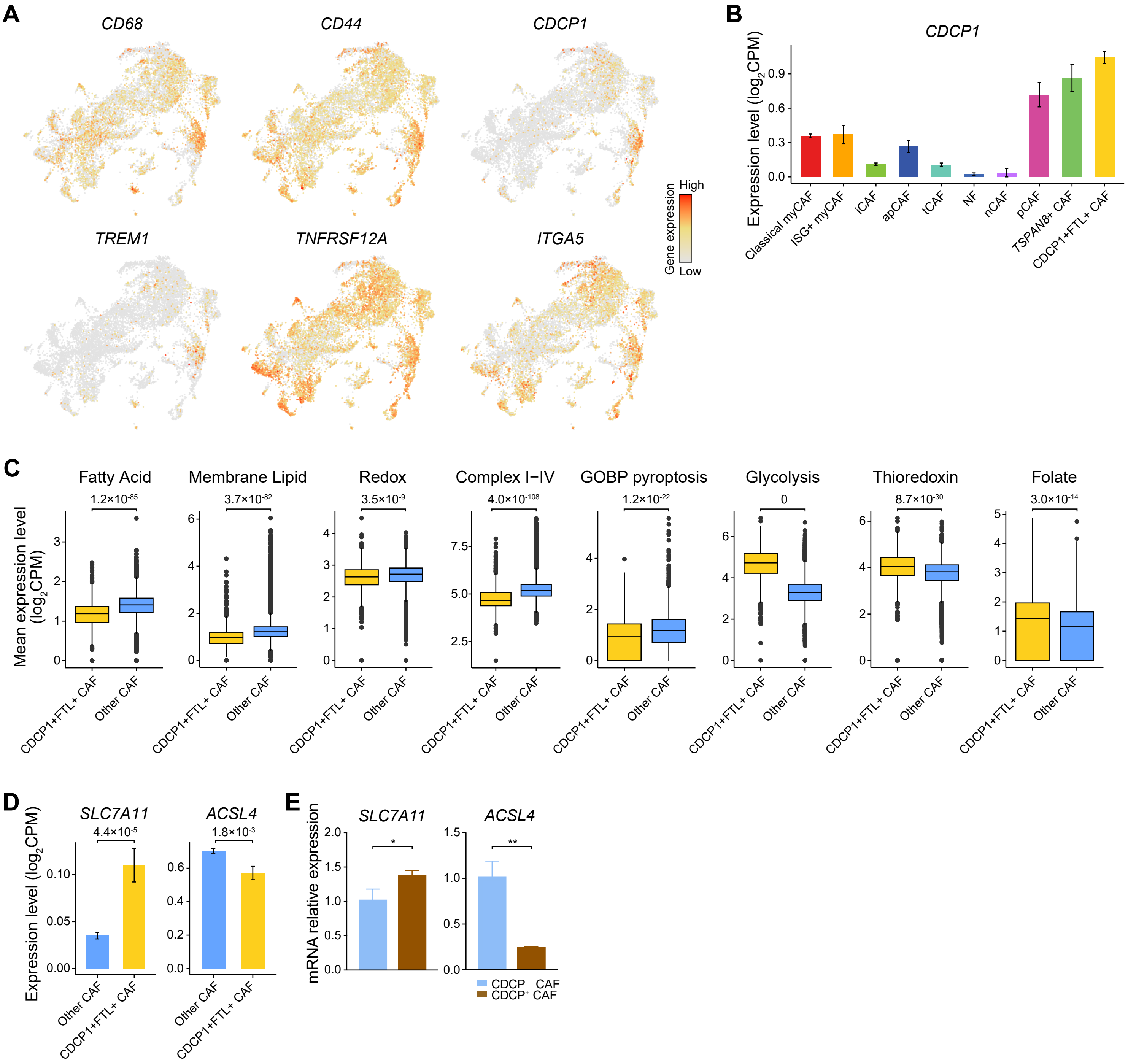
A
CD68
CD44
CDCP1
TREM1
TNFRSF12A
ITGA5
Gene expression
High
Low
B
CDCP1
Expression level (log2CPM)
0.0
0.3
0.6
0.9
Classical myCAF
ISG+ myCAF
iCAF
apCAF
tCAF
NF
nCAF
pCAF
TSPAN8+ CAF
CDCP1+FTL+ CAF
C
Mean expression level (log2CPM)
Fatty Acid
1.2×10-85
Membrane Lipid
3.7×10-82
Redox
3.5×10-9
Complex I−IV
4.0×10-108
GOBP pyroptosis
1.2×10-22
Glycolysis
0
Thioredoxin
8.7×10-30
Folate
3.0×10-14
CDCP1+FTL+ CAF
Other CAF
D
SLC7A11
4.4×10-5
ACSL4
1.8×10-3
Expression level (log2CPM)
Other CAF
CDCP1+FTL+ CAF
E
mRNA relative expression
SLC7A11
*
ACSL4
**
CDCP- CAF
CDCP+ CAF

Figure S3. Identification and functional role of $CDCP1^{+}FTL^{+}$ CAF in PDAC.

(A) UMAP plots showing the expression level of marker genes.

(B) Bar plots showing the expression level of *CDCP1* in CAF subtypes. Data are shown with the mean value ± SD.

(C) Boxplots showing the expression level of ferroptosis-related metabolic pathways. Two-tailed Student's *t*-test *P* values are indicated.

(D) Bar plot showing the expression level of ferroptosis-related genes *SLC7A11* and *ACSL4*. Two-tailed Student's *t*-test *P* value is indicated.

(E) mRNA expression of ferroptosis related genes *SLC7A11* and *ACSL4* of sorted $CDCP1^{+/-}$ CAFs. Two-tailed Student's *t*-test *P* value is calculated, $^{*}P < 0.05$, $^{**}P < 0.01$.

Figure S4

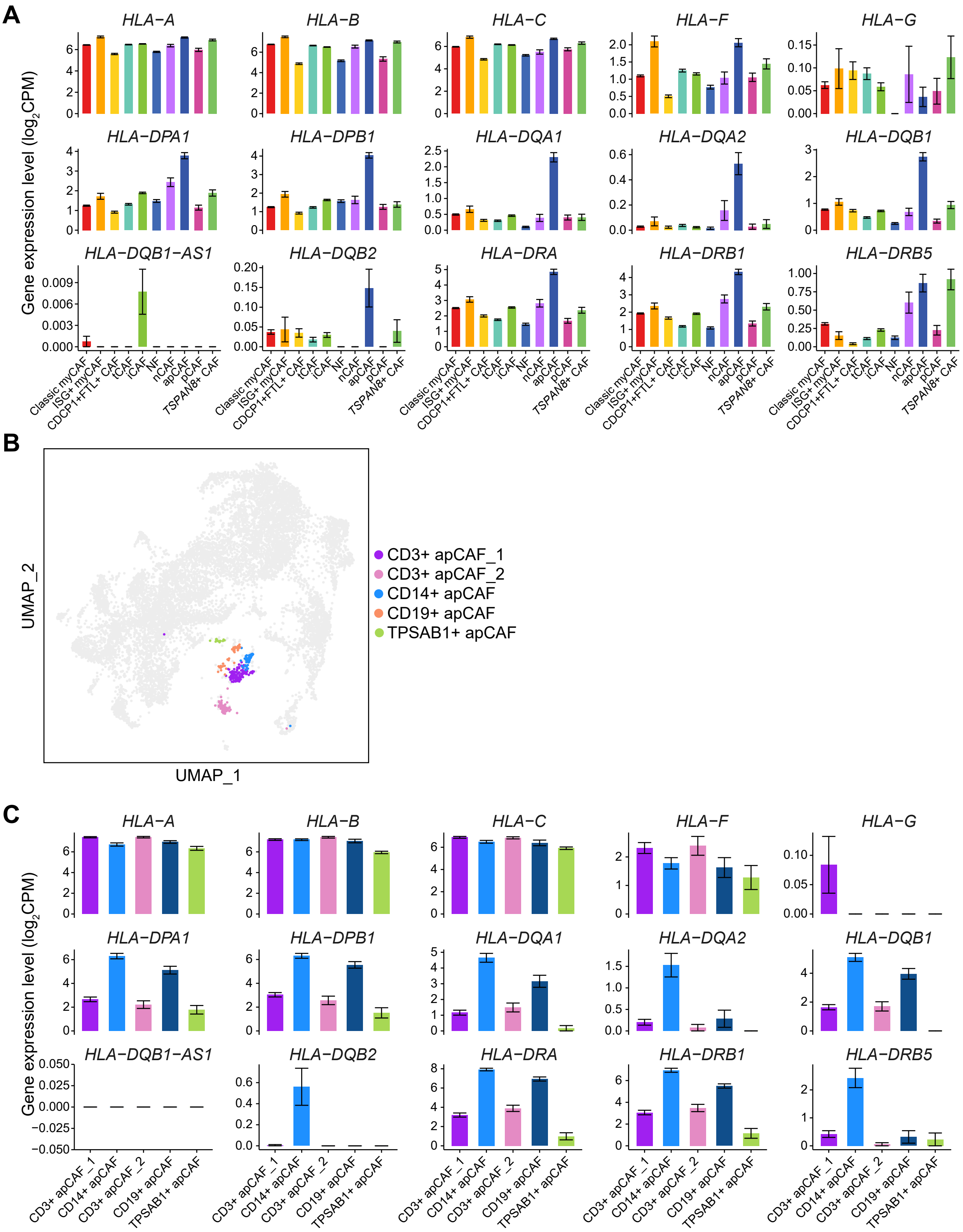

A
HLA−A
HLA−B
HLA−C
HLA−F
HLA−G
HLA−DPA1
HLA−DPB1
HLA−DQA1
HLA−DQA2
HLA−DQB1
HLA−DQB1−AS1
HLA−DQB2
HLA−DRA
HLA−DRB1
HLA−DRB5
Gene expression level (log2CPM)
Classic myCAF
ISG+ myCAF
CDCP1+FTL+ CAF
tCAF
iCAF
NF
nCAF
apCAF
pCAF
TSPAN8+ CAF
B
UMAP_2
UMAP_1
CD3+ apCAF_1
CD3+ apCAF_2
CD14+ apCAF
CD19+ apCAF
TPSAB1+ apCAF
C
HLA−A
HLA−B
HLA−C
HLA−F
HLA−G
HLA−DPA1
HLA−DPB1
HLA−DQA1
HLA−DQA2
HLA−DQB1
HLA−DQB1−AS1
HLA−DQB2
HLA−DRA
HLA−DRB1
HLA−DRB5
Gene expression level (log2CPM)
CD3+ apCAF_1
CD14+ apCAF
CD3+ apCAF_2
CD19+ apCAF
TPSAB1+ apCAF

Figure S4. Identification of apCAF subtypes and immune cells co-expressed CAF marker genes.

(A and C) Barplots showing the expression level of MHC-I and MHC-II genes in all fibroblasts (A) or apCAF subtypes (C). Data are shown with the mean value ± SEM.

(B) UMAP plot showing the distribution of apCAFs in all fibroblasts from normal and tumor tissues. The dot color indicates apCAF subtypes.

Figure S5

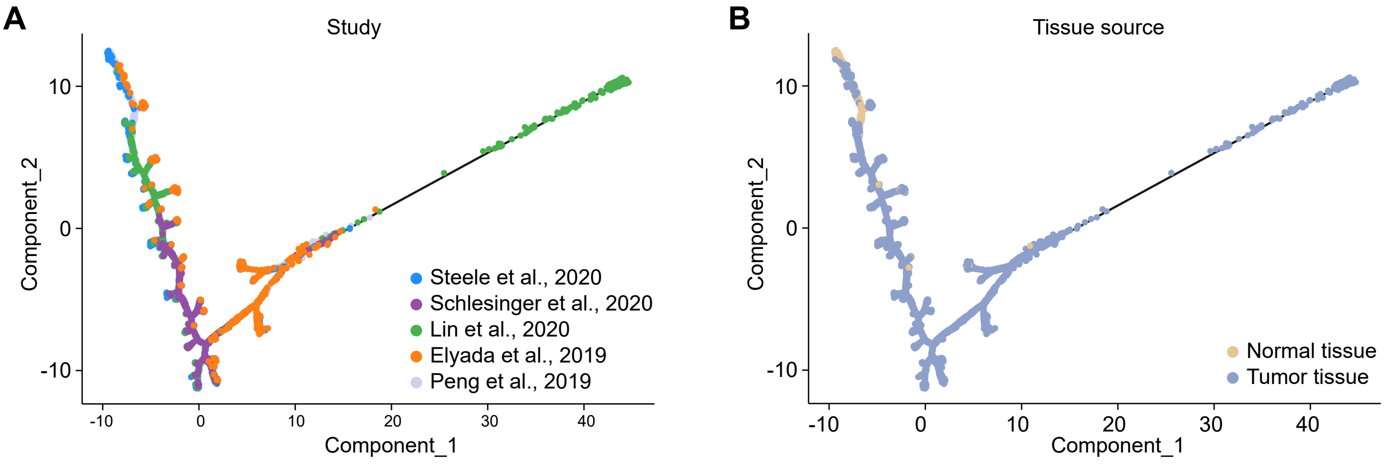
A
Study
Component_2
Component_1
Steele et al., 2020
Schlesinger et al., 2020
Lin et al., 2020
Elyada et al., 2019
Peng et al., 2019
10
0
-10
-10
0
10
20
30
40
B
Tissue source
Component_2
Component_1
Normal tissue
Tumor tissue
10
0
-10
-10
0
10
20
30
40

Figure S5. Pesudotime analysis of distinct CAF subtypes.

Pesudotime analysis showing the differentiated trajectory of distinct CAF subtypes. The dot color indicates data sources (A) or tissue sources (B).

Figure S6

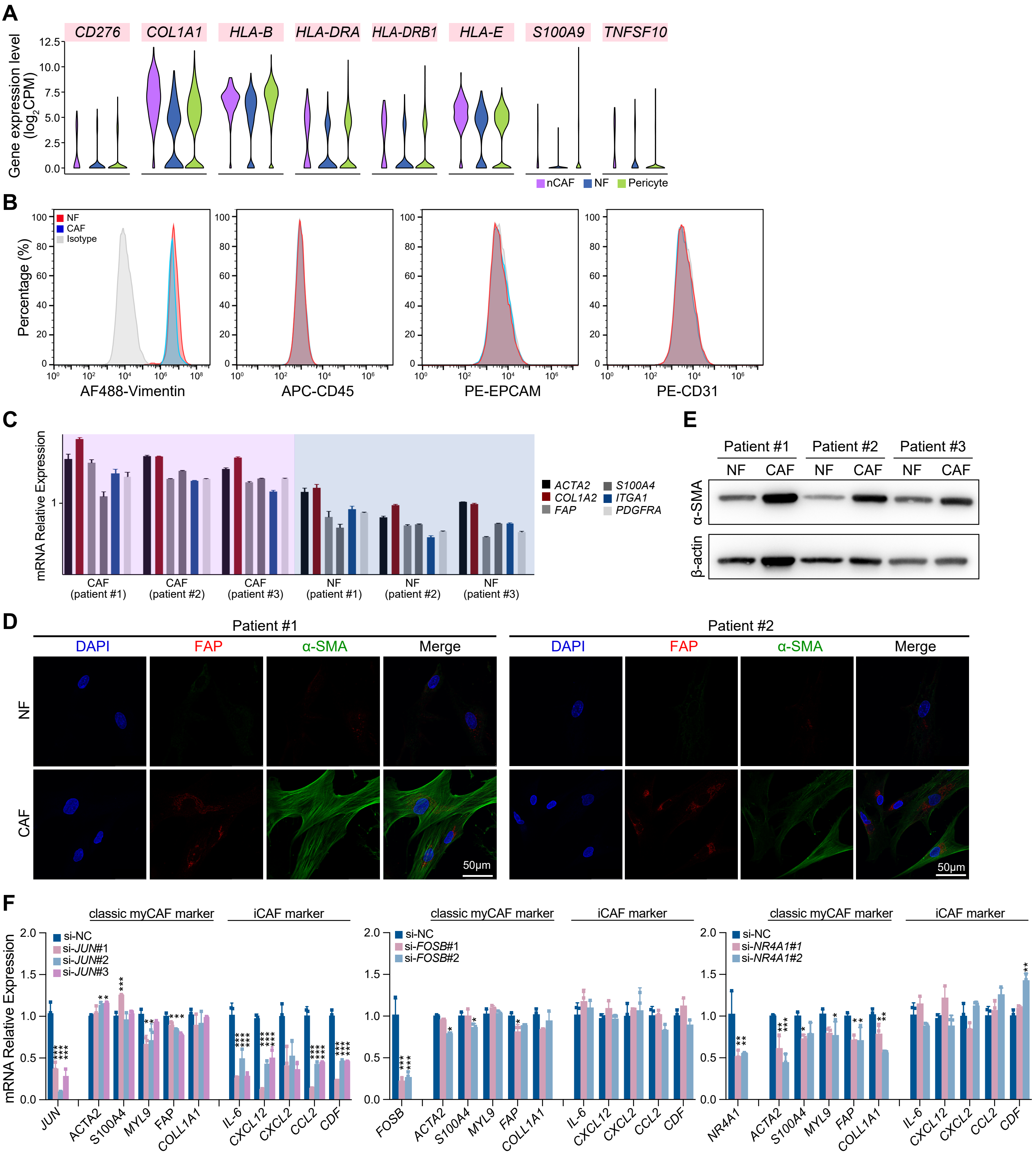
A
CD276
COL1A1
HLA-B
HLA-DRA
HLA-DRB1
HLA-E
S100A9
TNFSF10
Gene expression level (log_2 CPM)
nCAF
NF
Pericyte
B
NF
CAF
Isotype
Percentage (%)
AF488-Vimentin
APC-CD45
PE-EPCAM
PE-CD31
C
mRNA Relative Expression
ACTA2
COL1A2
FAP
S100A4
ITGA1
PDGFRA
CAF (patient #1)
CAF (patient #2)
CAF (patient #3)
NF (patient #1)
NF (patient #2)
NF (patient #3)
E
Patient #1
Patient #2
Patient #3
NF
CAF
α-SMA
β-actin
D
Patient #1
Patient #2
DAPI
FAP
α-SMA
Merge
NF
CAF
50μm
F
classic myCAF marker
iCAF marker
mRNA Relative Expression
si-NC
si-JUN#1
si-JUN#2
si-JUN#3
si-FOSB#1
si-FOSB#2
si-NR4A1#1
si-NR4A1#2
JUN
FOSB
NR4A1
ACTA2
S100A4
MYL9
FAP
COLL1A1
IL-6
CXCL12
CXCL2
CCL2
CDF

Figure S6. AP-1 members triggering the malignant phenotype conversion of NFs.

(A) Violin plots showing the expression level of representative genes in nCAFs, NFs, and pericytes.

(B) Flow cytometry indicating the expression of vimentin in human primary CAF and the absence of CD45, EPCAM and CD31.

(C) Bar plot showing the mRNA expression of representative genes in paired human primary PDAC CAFs and NFs. Data are shown with the mean value ± SEM.

(D) Representative of confocal microscopy images of FAP and α-SMA staining in paired human primary PDAC CAFs and NFs. Scale bar, 50 μm.

(E) Western blots showing α-SMA levels in paired human primary PDAC CAFs and NFs.

(F) Bar plots showing the mRNA expression of representative genes of classic myCAF and iCAF in human primary CAFs after knocking-down *JUN*, *FOSB*, and *NR4A1* by si-RNA. Two or three different siRNAs were used for avoiding off-target effect. For each group, $n = 3$ biological replicates. Data are shown with the mean value ± SD. *P* value by one-way ANOVA, $^{*}P < 0.05$, $^{**}P < 0.01$, $^{***}P < 0.001$, $^{****}P < 0.0001$.

Figure S7

A

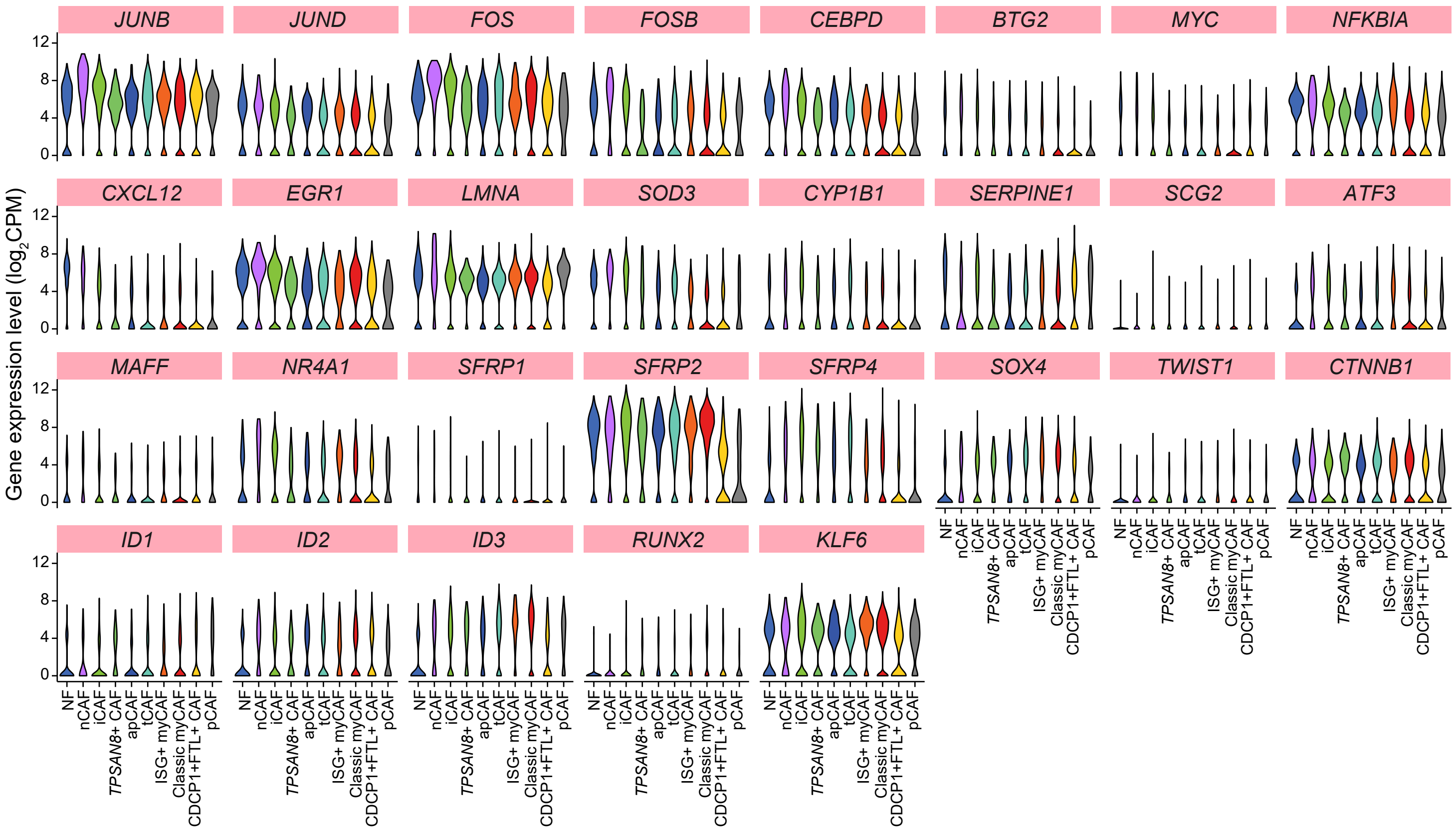
JUNB
JUND
FOS
FOSB
CEBPD
BTG2
MYC
NFKBIA
CXCL12
EGR1
LMNA
SOD3
CYP1B1
SERPINE1
SCG2
ATF3
MAFF
NR4A1
SFRP1
SFRP2
SFRP4
SOX4
TWIST1
CTNNB1
ID1
ID2
ID3
RUNX2
KLF6
Gene expression level (log_2CPM)
0
4
8
12
NF
nCAF
iCAF
TPSAN8+ CAF
apCAF
tCAF
ISG+ myCAF
Classic myCAF
CDCP1+FTL+ CAF
pCAF

B

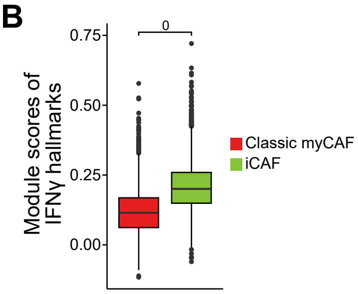
Module scores of IFNγ hallmarks
0.00
0.25
0.50
0.75
0
Classic myCAF
iCAF

Figure S7. IFNγ contributing to the fate transformation from classic myCAFs to iCAFs

(A) Violin plots showing the expression level of representative genes across CAF subtypes.

(B) Boxplot showing the module score of IFNγ hallmarks in classic myCAFs and iCAFs. The two-tailed Student's *t*-test *P* value is indicated.

Figure S8

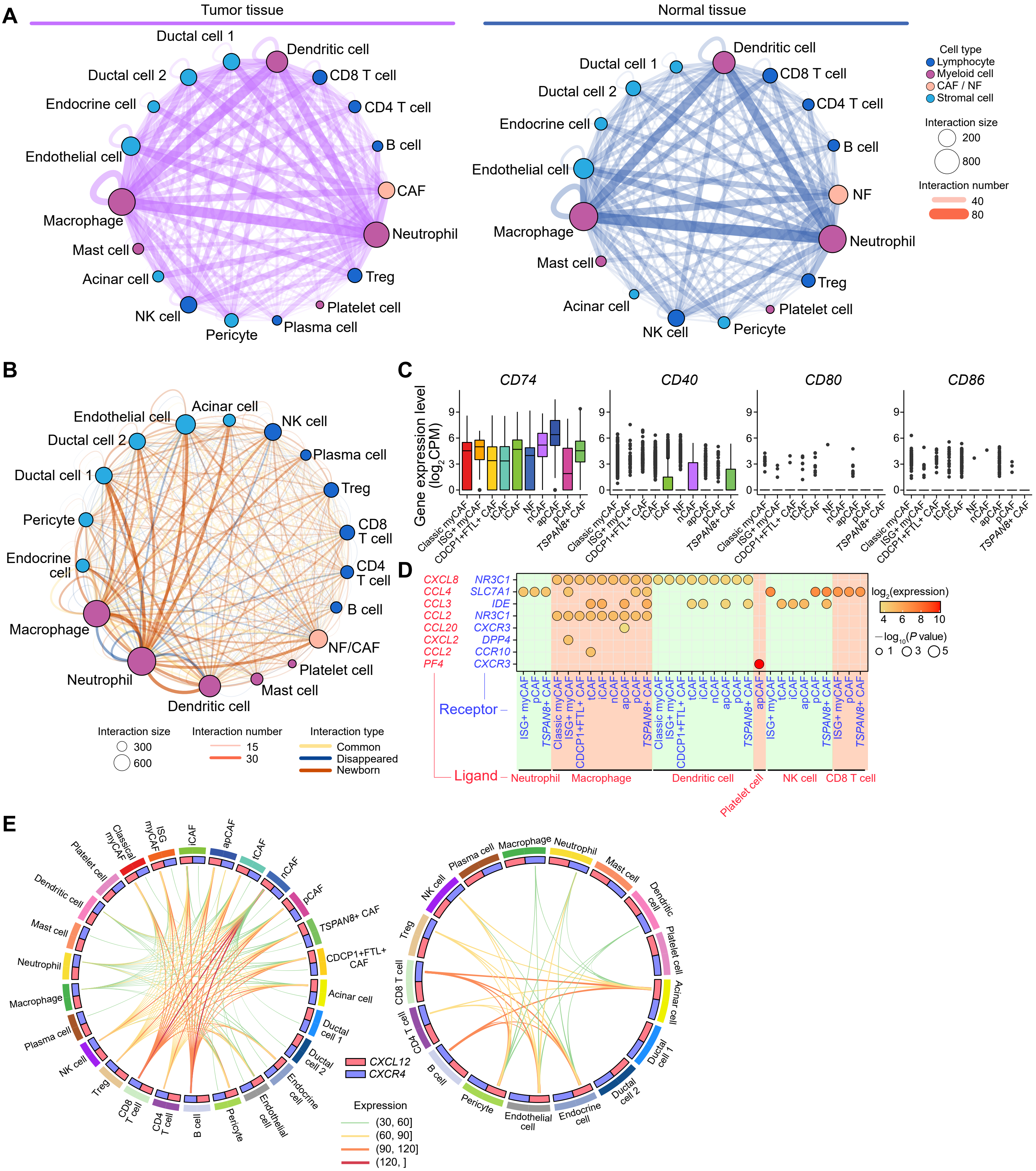
A
Tumor tissue
Normal tissue
Ductal cell 1
Dendritic cell
Ductal cell 2
CD8 T cell
Endocrine cell
CD4 T cell
Endothelial cell
B cell
CAF
NF
Macrophage
Neutrophil
Mast cell
Acinar cell
Treg
Platelet cell
NK cell
Plasma cell
Pericyte
Cell type
Lymphocyte
Myeloid cell
CAF / NF
Stromal cell
Interaction size
200
800
Interaction number
40
80
B
Acinar cell
Endothelial cell
NK cell
Ductal cell 2
Plasma cell
Ductal cell 1
Treg
Pericyte
CD8 T cell
Endocrine cell
CD4 T cell
B cell
Macrophage
NF/CAF
Platelet cell
Neutrophil
Mast cell
Dendritic cell
Interaction size
300
600
Interaction number
15
30
Interaction type
Common
Disappeared
Newborn
C
CD74
CD40
CD80
CD86
Gene expression level (log2 CPM)
Classic myCAF
ISG+ myCAF
CDCP1+FTL+ CAF
tCAF
iCAF
NF
nCAF
apCAF
pCAF
TSPAN8+ CAF
D
CXCL8 NR3C1
CCL4 SLC7A1
CCL3 IDE
CCL2 NR3C1
CCL20 CXCR3
CXCL2 DPP4
CCL2 CCR10
PF4 CXCR3
log2(expression)
4 6 8 10
−log10(P value)
1 3 5
Receptor
Ligand
Neutrophil
Macrophage
Dendritic cell
Platelet cell
NK cell
CD8 T cell
E
CXCL12
CXCR4
Expression
(30, 60]
(60, 90]
(90, 120]
(120,]

Figure S8. Cell-cell interactions between CAF subtypes and other cells.

(A) Cell-cell interaction network showing interactions between CAF subtypes and other cells in tumor (left) and normal (right) tissues. The dot color indicates the cell type, and the dot size indicates the interaction number of a given cell type. The line thickness indicates the interaction number of a given cell type pair.

(B) Cell-cell interaction network showing distinct types of interactions between CAFs (or NFs) and other cells. The line color indicates the interaction type, newborn interactions (tumor specific interactions) in red, disappeared interactions (normal tissue specific interactions) in blue, and common interactions (coexisted in tumor and normal tissues) in yellow. The dot color indicates the cell type, the dot size indicates the interaction number of a given cell type, and the line thickness indicates the interaction number of a given cell type pair.

(C) Boxplots showing the expression of costimulatory genes in NFs and distinct CAFs.

(D) Dot plot showing the expression level of ligand-receptor gene pair related with chemotaxis and cytokine within lymphocyte-CAF interactions. The dot color and size indicate the expression levels and statistical significance, respectively. Red words indicate the ligands (row) expressed in the corresponding cell type (column), and blue words indicate the receptors (row) expressed in the corresponding cell type (column).

(E) Circle plots showing *CXCL12-CXCR4* gene pair in pairwise cell types within tumors (left), or in pairwise cell types (exclude CAFs) within tumors (right). For a give line in this plot, the endpoint indicates the cell type expressing ligand *CXCL12*, and the other endpoint indicates the cell type expressing receptor *CXCR4*.
